# Magnitude and determinants of road traffic accidents in North Gondar Zone; Amhara Region, Ethiopia

**DOI:** 10.12688/f1000research.123111.1

**Published:** 2023-04-05

**Authors:** Abay Semegnew Molla

**Affiliations:** 1 Department of Civil Engineering, Institute of Technology, University of Gondar, P.O.Box:196, Gondar, Ethiopia

**Keywords:** Road traffic accidents, causality, injuries, property damage, negative impacts.

## Abstract

Background: Despite efforts that the government has made to minimize the number of casualties or injury levels in Ethiopia, road traffic accidents remain a severe health concern throughout the country and in the study area as well. This study aims to examine the primary contributing variables of road traffic accidents in the North Gondar zone, under 24 administrative.

Methods: The functional class of roads were directly examined on the site to evaluate the existing conditions of the roads which includes the geometric design constraints, functional class, daily traffic, and surfacing materials. Secondary source of data types, previously recorded road traffic accident information was collected from North Gondar zone traffic police departments. Road inventory information was collected from Gondar branch Ethiopian road authority under the specified horizon lines.

Results: The magnitude and frequency of traffic accidents caused by driver-related factors( age, sex, level of education, driving experience, ownership responsibility of a vehicle and driver responsibility, time and day-wise distribution of traffic accidents), vehicle-related factors (failures of mechanical parts and its service age), road related factors( geometric design and construction, inventory information, road surfacing materials), pedestrians, and environmental factors were critically analysed. The result shows that a higher proportion (67%) of traffic accidents are mainly due to driver behaviour related factors than other causative factors.

Conclusions: According to the results of the study, bad driving behaviour’s (exceeding the design speed, using a phone while driving, dangerous overtaking, excessive braking, loading above capacities, illegally parking out of public stations should be controlled to reduce the road traffic accident in the study area. In addition, successive up-to-date advanced computerized traffic accident safety training and recording systems should be given at least once a year through regional-based or at a central zone administrative zones for the traffic officers.

## Introduction

Asegie (2019) indicated that in Sub-Saharan Africa, particularly Ethiopia, where alternative modes of transportation are scarce, road transportation has served as an economic pillar, an inducer of commerce, trade, and traditions, as well as the growth of feelings, cultures, and people's socio-economic lives. This form of transportation has assisted the country's growth by facilitating the movement of agricultural and industrial commodities, as well as services, from one area to the next and to far-flung villages. Worku (2011) noted that road transportation is intended to create a network that connects a variety of infrastructures. Alternative contemporary forms of transportation are too expensive for developing countries such as Ethiopia, so the road transport business is vital. According to Gebrechristos (2014), road transportation has a foundation in the eradication of poverty and offers the country urban-to-urban and urban-to-rural connectivity. The relevance of rural road infrastructure has been recognized in both the country's general development policies and a multitude of sector initiatives ( Emmenegger, 2012).

Even if road transportation is a need for subsistence, its traffic accidents are one of the world's most serious problems, including fatalities and property loss. The most prevalent causes of traffic accidents are human factors, vehicle factors, and road conditions ( Rolison *et al.*, 2018). Tiruneh *et al.* (2014) studied that patients who come to Addis Ababa Black Lion Hospital for treatment are more likely to be involved in a road accident, as evidenced by the results of inebriated driving. A six-month institutional-based cross-sectional study on patients at the University of Gondar Comprehensive Teaching and Referral Hospital was conducted to examine road traffic accidents and related factors. Based on the study, results show that there was a high traffic accident among traumatized patients ( Honelgn & Wuletaw, 2020).

A community-based cross-sectional research study with a total sample size of 422 people was conducted in Chuko Town. According to the study, poor road conditions were the main cause, accompanied by motorbike overspeeding ( Tegegne, 2020). Shamenna *et al.* (2021) analysed the spatial distribution of road traffic accidents in Hawassa City. As per this investigation, sloppy driving, refusing to secure pedestrians' priority, driving too fast, and failure to give each other space by the drivers are the causes of traffic accidents.

Tadege (2020) conducted in Finoteselam town to determine the rate of traffic accidents. In fatal traffic accidents, drivers' lack of driving expertise, inadequate educational level, age of the driver, weekend time driving, and driving in the summer season were listed as important causes of the problem. Using six years of data, Tullu *et al.* (2013) studied the characteristics of police-reported road traffic accidents in Ethiopia. The researcher observed that there is a higher proportion of pedestrians. The collisions happen during daylight, affect men, and happen in the center of the roads. Rollover on a road tangent, failure to follow pedestrian priority, and overspeeding are all factors that contribute to the traffic accident.

Hordofa *et al.* (2018) cited that road traffic fatalities are seen as a man-made calamity in Ethiopia. The Ethiopian National Road Safety Office claims a fatality rate of 114 per 10,000 cars per year. However, due to an inefficient reporting system, the true figure might be higher. Aside from the loss of life, traffic accidents cost an extra amount of money for medical treatments, physical discomfort, permanent impairment, and travel stress. It also has an impact on patients’ household income, lowering their quality of life and the national economy as well.

As a result of rapid motorization without adequate regulation, rapid population growth and expansion of the road network, and poor attitudes and safety culture among road users, the likelihood of a road traffic accident is increasing in Ethiopia. Although there are limited activities geared towards combating the problem, they are insufficient by any standard relative to the worsening situation. Road safety management tends not to receive due consideration because of the non-availability of research carried out in the area. Hence, creating a sound road traffic accident database is a very important tool to identify the major causes of the accidents and work towards improvements. In general, without the basis of a dependable traffic accident data recording system, there could be no effective road safety work in our country, specifically in the study area.

Road accidents and traffic management are issues of vital concern to road authorities. Detecting the risk factors for accidents can contribute to making a road more efficient, safe, and comfortable. This study focuses on issues relating to road surface conditions, traffic facilities, and road users' conduct that lead to traffic accidents. Roads from Gondar town adjacent to Wereda town in North Gondar administrative boundaries were selected to serve as the case study. A field survey of the current traffic accident conditions was conducted on the selected road networks. Previous accidents, road surface defects, and insufficient traffic control facilities that are directly connected to road safety were visually and technically inspected in the analysis. This study relies on the identification of the basic causes of problems along with the selected road networks for effective recommendations and safety management.

## Methods

The study was conducted in the North Gondar administrative zone, located in the Amhara region, 745 kilometres north of Addis Ababa. It includes 24 weredas in the zonal study area. The total population was estimated at 2,929,628 ( CSA, 2007). This study was a descriptive type of research in which both qualitative and quantitative types of data. It focuses on the assessment and analysis of the road traffic accident causative factors in the North Gondar zone administrative boundaries (the roads bounded in 24 werda’s). Secondary sources of data were used to achieve the intended objective of the research. The main objective of this study was to assess and analyse the major contributing factors to road traffic accidents and their effects on life lost, injuries, and property damage in the specified study area. A five year data which includes road user’s characteristics, vehicle, road and environmental factors in relation to the frequency and intensity of road traffic accidents collected from north Gondar zone police departments and road functional database management systems obtained from Gondar branch Ethiopian road authority are the sources of data for the analysis.

The five years of police report data (2015 to 2019) for this research study area served as the data sources. Excel (version 2013) and SPSS (version 2020) were used as statistical tools for the data analysis.

## Results

### Age Distribution

There was an incidence of highest records (34 percent and 24.85 percent of personal aggregate effects and property damage) out of the 91 and 507 personal injuries and property damage that occurred in those five years. Furthermore, the age group (19-30 years) had the highest record of person and property damage statistics (56 percent and 62 percent, respectively) than the other age groups.

Table 1 illustrates the driver's age and the types of incidents. Young drivers between the ages of 19 and 30 are the most susceptible, with a total of 598 accidents (56.7 percent) compared to older age drivers (>50 years). It is because younger age groups engage in riskier activities and have less driving experience.

**Table 1.** Age level, type and number of road traffic accident records.

Years	Drivers’ age	Life lost	seriously injury	slightly injury	Property loss	Aggregate damage	Aggregate damage (%)
2015	<18	0	0	1	1	2	0.3
	19-30	31	23	22	17	93	15.6
	31-50	12	5	4	13	34	5.7
	>50	3	1	0	0	4	0.7
	Undetermined	12	8	4	1	25	4.2
2016	<18	5	3	2	1	11	1.8
	19-30	38	12	16	16	82	13.7
	31-50	11	1	3	3	18	3.0
	>50	1	0	1	0	2	0.3
	Undetermined	23	6	2	3	34	5.7
2017	<18	4	2	0	0	6	1.0
	19-30	37	13	16	13	79	13.2
	31-50	8	3	1	3	15	2.5
	>50	0	0	0	0	0	0.0
	Undetermined	19	4	5	1	29	4.8
2018	<18	2	0	0	0	2	0.3
	19-30	28	8	8	5	49	8.2
	31-50	7	3	6	2	18	3.0
	>50	1	0	0	0	1	0.2
	Undetermined	12	3	2	1	18	3.0
2019	<18	2	0	1	1	4	0.7
	19-30	15	6	10	5	36	6.0
	31-50	11	1	0	2	14	2.3
	>50	1	1	0	0	2	0.3
	Undetermined	12	5	0	3	20	3.3

### Driver’s Level of Education

**Figure 1.**
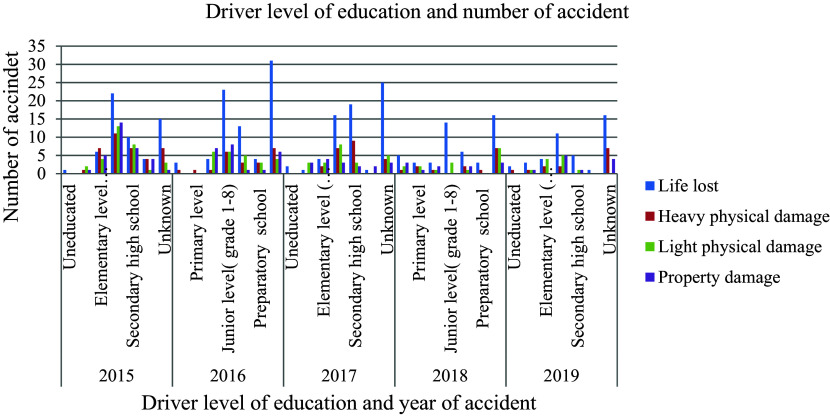
Driver level of education, type and number of road traffic accident records.

Figure 1 shows that, in addition to age, the educational level has a significant impact on the incidence of road traffic accidents. We believe that better-educated drivers pay more attention and are more concerned about reducing the risk of a road traffic collision than less educated drivers. As a result, a driver's education provides the information, abilities, and attitudes necessary for vehicle safety, both as a driver and as a pedestrian.

According to the driving license regulations in Ethiopia, a driver's license can be acquired after completion of junior high school. The highest (29.4 percent) out of 602 aggregate accidents recorded by junior level completed drivers than the others. This indicates that the occurrence of accidents is related to the poor quality of training and the unlawful approval of driving license systems.

### Driver Experience

**Figure 2.**
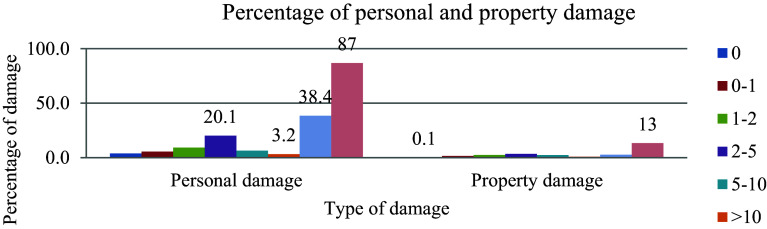
Driving experience and percentage of damages.

Figure 2 illustrates the driving experience and percentage of damages. Driving a vehicle for an extended period and embracing the behaviour of a car helps to minimize the occurrence of road traffic accidents. Inexperience is a flaw that can lead to mistakes in operation.

Out of the 685 cumulative effects of aggregate damage, the highest (87%) of damage was recorded in a driver without owning approved licenses. This indicates that there is a weak control system of driver’s licenses at traffic regulation offices.

### Drivers’ Ownership Responsibility

**Figure 3.**
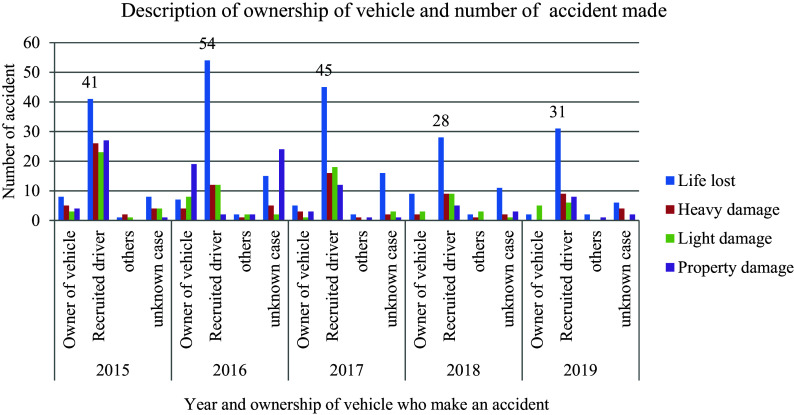
Vehicle ownership responsibility and number of damages.

Figure 3 shows the number of damages and the responsibilities of the vehicle's owner. The highest (63.2 percent) of 622 road traffic accidents occurred with a hired driver rather than the vehicle's owner. In contrast to the employed driver, the owner causes an average traffic accident of 14.6%. This is the fact that the owner of a commercial vehicle who is using great caution in limiting vehicle speed, increases the likelihood of a traffic collision.

### Vehicle Categories

**Figure 4.**
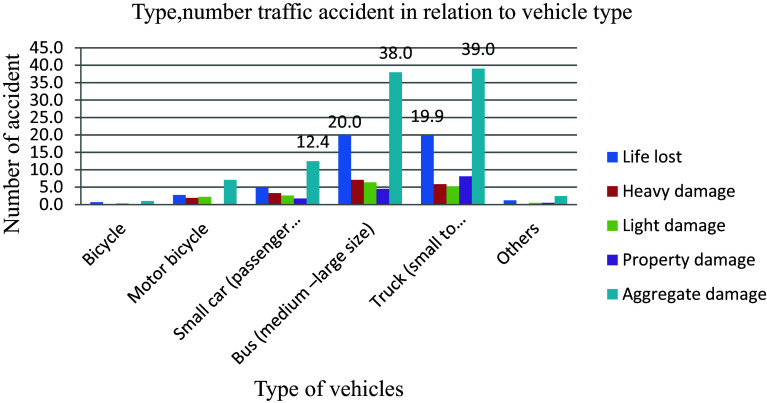
Vehicle catagories and number of road traffic accident.

When a vehicle collides with other vehicles, a pedestrian, an animal, road curbs, existing buildings, or any immovable barrier such as a tree, or electric pole, a building causes injury, death, and property damage. Trucks are a regular sight on the country's roadways, these vehicles are a vital element of the economy, transporting commodities from one location to another to fulfil the demands of the country's rapidly rising population. Large vehicles, while necessary for a society's existence, can pose a high risk to other small vehicles, especially when truck drivers are careless. Truck accidents of all kinds are responsible for some of the worst roadside destruction. Trucks (small to articulated) and buses (medium to large size) were involved in the highest (39 and 38) percent of the 579 total road traffic accidents shown in Figure 4. This is because truck vehicles are heavier and larger than passenger vehicles, and hence there is a higher risk of a traffic collision.

### Vehicle Mechanical Parts Failure and Service Age

**Figure 5.**
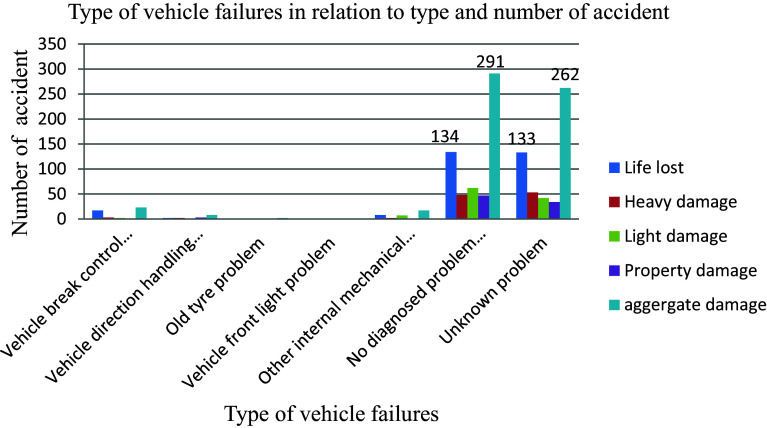
Vehicle mechanical parts failures and number of road traffic accident.

The vehicle mechanical issues problems cause road traffic accidents. The issues of vehicle mechanical parts are tire problems, blowouts and skidding due to worn tread, brake issues, slow brake time and brake failure, steering issues, such as pulling or loss of power steering, seatbelts that don't work, brakes that fail, airbags that injure, tires that have defects, or defective accelerators that can be inadvertently triggered. Out of 603 aggregate road traffic accidents recorded the highest (48.3% has come about with any diagnosed mechanical failures than any defined internal and supportive parts of vehicle problems as indicated in Figure 5.

**Figure 6.**
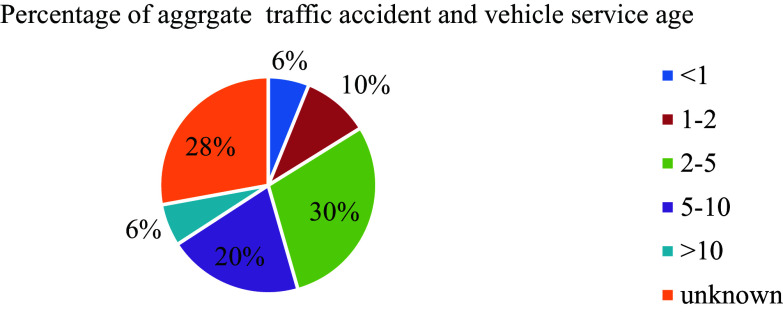
Percentage of aggregate damage and vehicle service age.

In parallel to this, the vehicle service age has also an effect on road traffic accidents. In Ethiopia even though the population of motorized vehicles is increased from time to time, it has no approved rule showing the vehicle service age limits which leads to traffic accidents likewise to other developed countries' experiences. Out of 588 aggregate road traffic accidents recorded the highest (29.4% and 27.9%) was committed by vehicle service years of (2-5) years and unknown registered age vehicle groups respectively than any other service age categories as shown in Figure 6. This is due that the country does not well a defined database showing the vehicle service age.

### Road Surfacing Material, Functional Class and Location

Table 2 shows road inventory information for this study analysis. The table includes the names of road segments, length of the stretches (km), Ethiopian road authority road identification reference, road class, the annual average daily traffic (AADT), the type of surfacing materials, and design code standards of the selected analysis roads, asphalt-covered surfacing material, and DC-4 to DC-6 design code standards, having an average annual AADT of 84 to 1533.

**Table 2.** Road inventory information of the selected analysis roads in central Gondar zone.

No	Route/Road segment	Stretch km	Road No	Road class	AADT	Surface type	Design standard
1	Bahirdar-Gondar						
1.1	Woreta-Maksegnit	74	A3-9	Trunk	1533	Asphalt	DC-6
1.2	MaksegnitAzezo	28	A3-9		1533	Asphalt	DC-6
1.3	Airport- Gondar	13	A3-10		591	Asphalt	DC-5
1.4	Gondar by pass	13					DC-6
2	Gondar Humera	290		Main Access	475	Asphalt	
2.1	Gondar Jun-Museibameb	40	C35-1		475	Asphalt	DC-5
2.2	Museibameb -Bebew River	58	C35-1		475	Asphalt	DC-5
2.3	Bebew River -Angereb River	22	C35-1		475	Asphalt	DC-5
2.4	Angereb River -Dansha	45	C35-1		475	Asphalt	DC-5
2.5	Dansha - Humra	80	C35-1		475	Asphalt	DC-5
2.6	Rawian-Lugdi	45					DC-5
3	Azezo Junction-Metma	185		Main Access	1068	Asphalt	
3.1	Azezo Junction- Bohona	35	C34-1		1068	Asphalt	DC-5
3.2	Bohona- Negadiebahir	65	C34-1		1068	Asphalt	DC-5
3.3	Negadiebahir- Shehdi	45	C34-1		1068	Asphalt	DC-5
3.4	Shehdi-Metma	40	C34-1		1068	Asphalt	DC-5
4	Shehdi- Shawera- Seraba	462		Feeder		Gravel	
4.1	ShehdiGelgo	125	E304-1		371	Gravel	DC-4
4.2	Gelgo-Shawera	157			284	Gravel	DC-4
4.3	Serab-Delgi-Shawra	100	E303-1		84	Gravel	DC-4
4.4	Airport-Gorgora	52	E33-1		192	SD	DC-4
4.5	Chuhait-Delgi	28			170	Gravel	DC-3
5	Gondar -Buya River	199		Link			
5.1	Gondar-A/Giorgise	40	B30-2		512	Asphalt	DC-6
5.2	A/Giorgise-Debark	60	B30-2		462	Asphalt	DC-6
5.3	Debark -Dagusit	24	B30-2		312	Gravel	DC-4
5.4	Dagusit-Unzo	35	B30-2		312	Gravel	DC-5
5.5	Unzo-Adirkay	20	B30-2		312	Asphalt	DC-5

**Figure 7.**
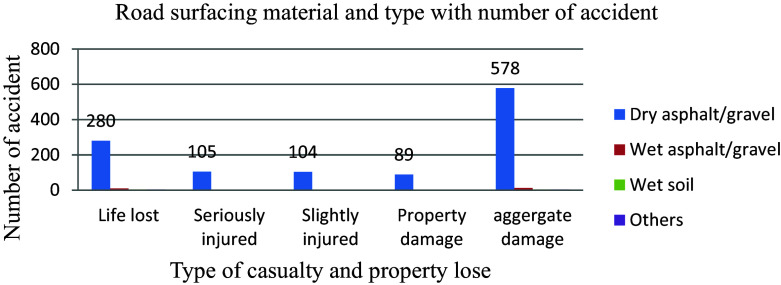
Aggregate damage and road surfacing material.

Pavement degradation and faults cause sliding, driving off tracks, inappropriate manoeuvring to avoid road imperfections, and extended driver braking distance, which requires immediate care from road and traffic authorities. Besides the type of surfacing materials, dry and wet conditions of the road, poor surfacing, macro, and micro-texture could lead to hydroplaning and inconsistency in tire pavement contact and also a reduction in tire gripping the pavement, which eventually causes accidents. Throughout the study year, 578 (96.2 percent) of the 601 aggregate road traffic accidents occurred on dry asphalt/gravel road surfaces rather than the most common wet soil conditions. As shown in Figure 7, the impacts of this road traffic collision are insignificantly connected to road conditions rather than human causes.

**Figure 8.**
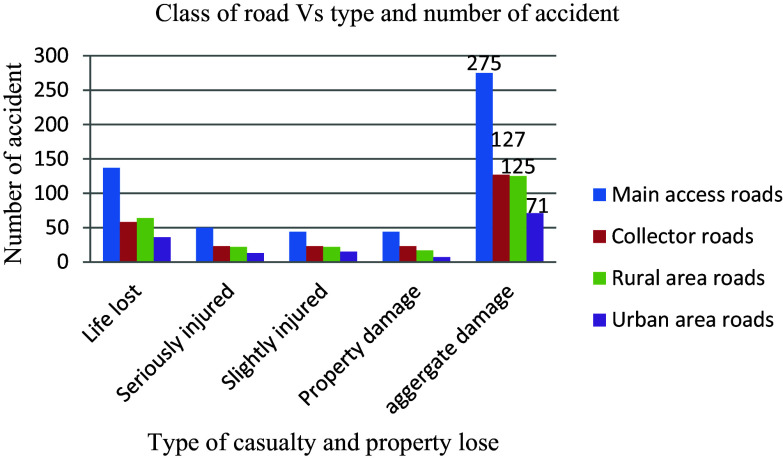
Aggregate damage and road functional classes.

Traffic safety is influenced by the different types of roadways. Although it is difficult to completely avoid traffic collisions, it is feasible to reduce the severity of their consequences by making roads and cars safer and drivers more cautious. Furthermore, by having a better understanding of the influence of different functional classifications of road, the risk may be lowered. Out of the 598 recorded casualties and property losses, in the main access roads with 475 to 1068 annual average daily traffic (AADT) had the highest 275 (46%) than any other functional class, as shown in Figure 8. This indicates that an increase in daily traffic volume above the route's capacity has a negative influence on road traffic accidents.

**Figure 9.**
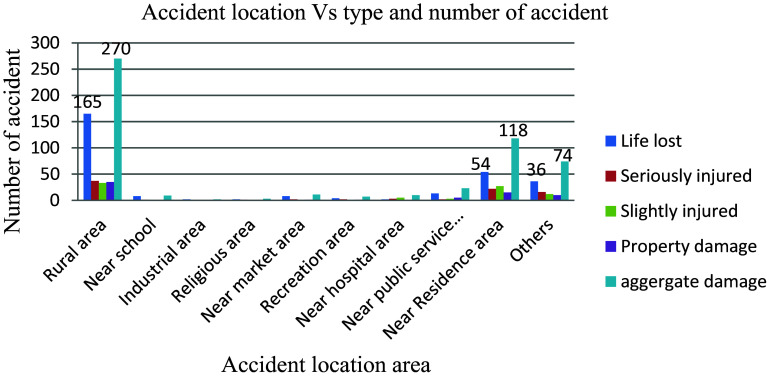
Aggregate damage and accident prone location.

The location and road stretch have an adverse effect on road traffic accidents. Figure 9 shows that, of the 527 total accidents recorded, 270 (51.2%) and 118 (22.4%) occurred in rural and residential areas, respectively, compared to other areas. This indicates that in rural areas, the local residents, especially farmers, do not have sufficient awareness and consciousness to protect themselves from road traffic accidents. In contrast, there are many young kids in front of schools who have limited awareness of road traffic accidents, vehicle speeds, sidewalk usage, recognizing pedestrian crosswalks, and other behaviours that might help lower the risk of a road traffic accident.

### Weather Condition and Day Time Situation

Through visual impairments, precipitation, severe winds, and temperature extremes, weather has an influence on driving capabilities, vehicle performance (traction, stability, and maneuverability). As shown in Table 3, the majority of weather-related crashes (547, or 91.9 percent) occur during visible day light (539, or 91.2 percent).

**Table 3.** Aggregate damage and weather condition.

Weather condition	Life lost	Seriously injured	Slightly injured	Property damage	aggregate damage	Aggregate damage (%)
Normal weather condition	275	86	95	83	539	91.2
Foggy	1	0	0	1	2	0.3
Cloudy	0	1	0	2	3	0.5
Light raining	1	4	1	2	8	1.4
Water flood (heavy rain)	1	0	0	0	1	0.2
Windstorm	0	0	0	0	0	0.0
Dust sky coverage	2	1	1	0	4	0.7
Warm climate	13	8	7	3	31	5.2
Bone chilling coldness	1	0	0	0	1	0.2
Others	1	1	0	0	2	0.3

### Time and Day Wise Distribution

**Figure 10.**
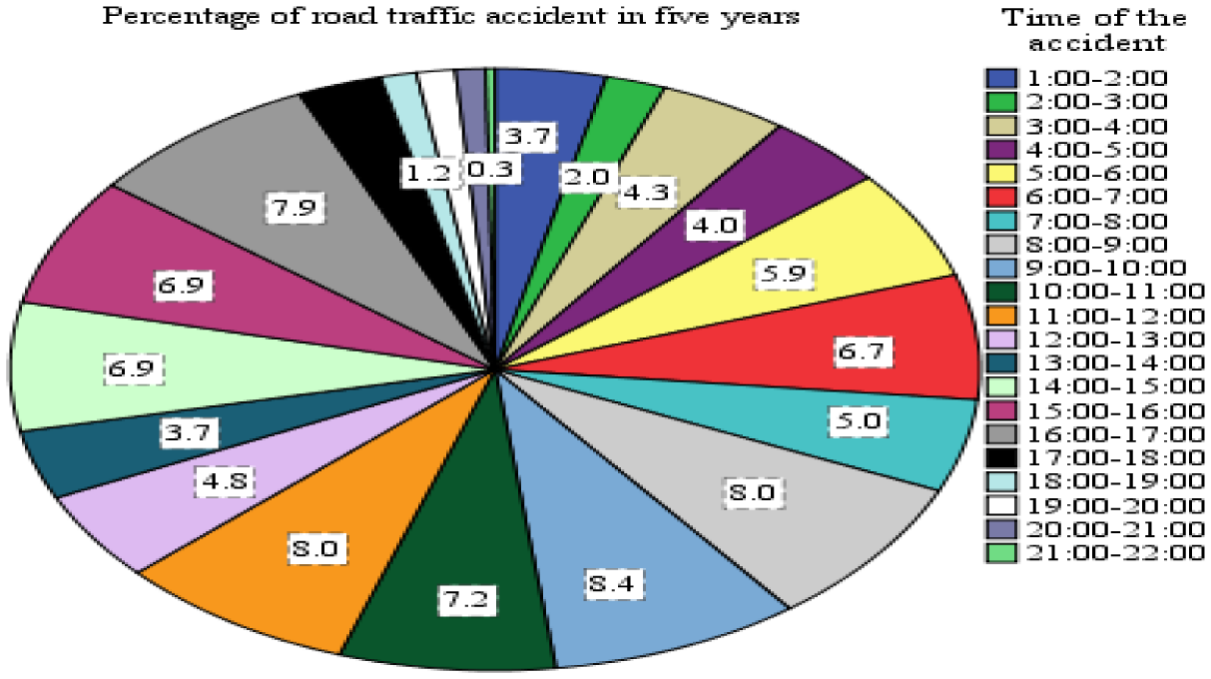
Percentage of traffic accident and time wise distributions.

Figure 10 shows the time-wise distribution of road accidents in the study area. It reveals there is a substantial variation in road traffic accidents during different times of the day. Out of 598 total recorded aggregate accidents, the highest (31.6% and 22%) were observed during the time intervals of 8:00 to 12:00 and (15:00 to 17:00) respectively. Hence, the highest amount of traffic flow was observed in the daytime rather than in the night, since a higher percentage of traffic accidents were recorded.

**Figure 11.**
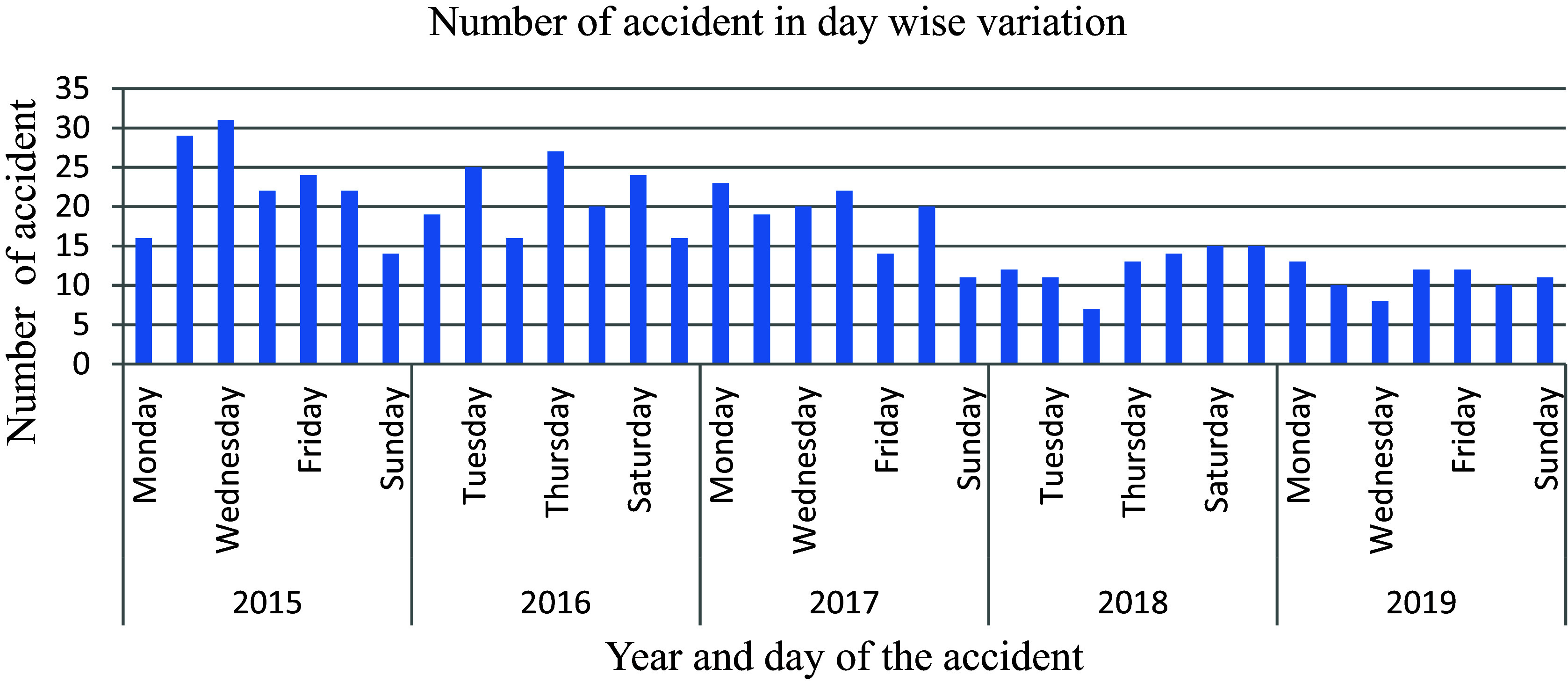
Number of road traffic accidents in day-wise variations.

As shown in Figure 11, there was less (2%) fluctuation among the days (Monday to Saturday) than there was on Sunday among the 597 traffic accidents registered in the police department. There have been fewer (11.2 percent) road traffic accident recordings since this date. This shows that there is less commercial vehicle traffic flow and passenger movement on Sundays, and so fewer accidents were seen.

### Category of Casualty

**Figure 12.**
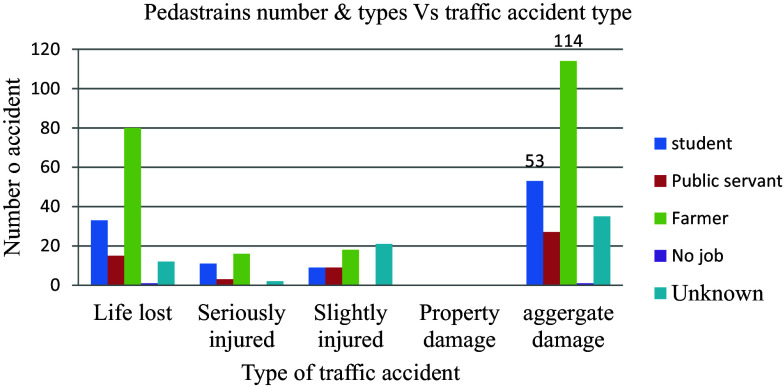
Aggregate damage with the type of casualty.

The incidence of road traffic injuries was 89 (38.7%) of the total of 230 aggregate damage that occurred. The majority of these victims were farmers, which accounted for 114 (49.6%), followed by students, which constituted 53 (23%) more victims than any other category of people as indicated in Figure 12.

## Discussions

In Ethiopia, including the study area, the basic accident data itself is collected by only the police and is recorded in a notebook using a locally developed standard format. In most cases, this takes place as a result of attending a road accident, but there are occasions when the accident is reported after the event at a police station. In this case, the missing of valuable information about the traffic accident's causative factors leads to misinterpretation of recommended actions to be taken. So, there should be a formal, centrally developed traffic accident recording standard in Ethiopia that may be adopted from developed countries having their systems automated. This will provide valuable information for academic issues and research.

In place of manual recording traditions, there should be an automated computerized web-based database recording system that works through the country regions also applicable in the study area, which will enable the traffic police offices and other road safety stakeholders to get summarized online traffic accident information at various levels easily and quickly. This can be done cooperatively with the federal police commission's central traffic accident analysis department and global consulting agencies that have good exposure to traffic database management systems. The system may include different subsystems for administrative and accident registration to meet its core functionalities, which are managed in different local languages.

As per the investigation, the cooperation between the concerned sectors which includes the road transport, traffic police departments, and the Ethiopian road authority and respective construction and housing development offices is low even though the traffic accident impact is high. In this case, a uniform centralized rule should be dispatched from the Amhara regional bureau to avoid the disparities interaction in this sector for traffic accident reduction.

A Higher proportion (67%) of traffic accidents are mainly due to driver behaviour-related factors than other causative factors. In this case, bad driving behaviours such as tailgating (driving too closely behind another car), exceeding the design speed, using a phone while driving, failing to indicate when turning directions, dangerous overtaking, excessive braking, a lack of understanding of roundabouts, loading and seating above the specified loading capacities and number of chairs, illegally parking and loading out of public stations.

A successive traffic accident safety trainings supported by up-to-date advanced computerized control and recording systems will be given at least once a year through regional-based or at a central zone administrative zones for the concerned traffic officers.

The traffic police officers should take higher responsibility to reduce the traffic accidents impacts in the area. If possible, it is better to stop an illegal communication with commercial vehicle drivers for the sake of their personal business enhancement. They should also give priority to passenger’s safety and worry about to human life lost. This should be managed through scheduled panel discussions with traffic officers and annual rewards.

Currently in the study area, annual traffic accident day celebrations, student traffic police formation and trainings, and pedestrian side keeping are practiced, and this should be promoted and continued further to increase traffic accident safety. In general, every individual, private sector government, and non-governmental organization should collaborate and play their respective roles to achieve an effective zero percent valuation of road traffic accidents in this area.

## Conclusions

Ethiopia, including the study area, is in the midst of a major road safety crisis. Thousands of people who work on the roads are slain every year. The prevalence of road traffic accidents has increased even though the government has given special attention to reducing its effect.

A descriptive type of research, in which both qualitative and quantitative types of data from secondary sources were used, was collected from previous traffic police reports. This kind of data collection was made by collecting the previous five years police report road traffic accident (2015–2019) in North Gondar zone police departments and functional road information from Gondar branch Ethiopian road authority offices.

The study focuses on analysing the frequency of traffic accidents caused by driver-related factors including age, sex, level of education, driving experience, ownership responsibility of a vehicle and driver responsibility, time and day-wise distribution of traffic accidents, vehicle-related factors like failures of mechanical parts of the vehicle and its service age, road geometric design and construction considerations like road inventory information, road surfacing materials, pedestrians, and environmental factors.

## Data availability

Zenodo. Magnitude and determinants of road traffic accidents in North Gondar Zone, Amhara Region, Ethiopia. DOI: https://doi.org/10.5281/zenodo.6948625 ( Molla, 2022).

This project contains the following underlying data:

- Number and types of a road traffic accidents in relation to road and road user, environmental and time related and vehicle related factors

Data are available under the terms of the Creative Commons Zero “No rights reserved” data waiver (CC BY 4.0 Public domain dedication).
