# Magnitude and determinants of road traffic accidents in North Gondar Zone; Amhara Region, Ethiopia

**DOI:** 10.12688/f1000research.123111.2

**Published:** 2024-05-24

**Authors:** Abay Semegnew Molla

**Affiliations:** 1 Department of Civil Engineering, Institute of Technology, University of Gondar, P.O.Box:196, Gondar, Ethiopia

**Keywords:** Road traffic accidents, causality, injuries, property damage, negative impacts.

## Abstract

**Background:** Home-based rehabilitation exercise following Total Hip Arthroplasty (THA) shows similar outcomes compared to supervised outpatient rehabilitation exercise. Little is known about patients’ experiences with home-based rehabilitation, and this study aimed to investigate patient-perceived facilitators and barriers to home-based rehabilitation exercise and general physical activity after THA.

**Methods:** Semi-structured interviews of qualitative design were conducted with 22 patients who had undergone THA and who had performed home-based rehabilitation exercise. The study took place in a regional hospital in Denmark between January 2018 and May 2019. Data was analyzed using an interpretive thematic analysis approach, with theoretical underpinning from the concept ‘conduct of everyday life’. The study is embedded within the Pragmatic Home-Based Exercise Therapy after Total Hip Arthroplasty-Silkeborg trial (PHETHAS-1), which aims to quantitatively investigate recovery outcomes after a home-based rehabilitation exercise program.

**Results:** The main theme, ‘wishing to return to the well-known everyday life’, and the subtheme ‘general physical activity versus rehabilitation exercise’ were identified. Generally, participants found the home-based rehabilitation exercise boring but were motivated by the goal of returning to their habitual conduct of everyday life and performing their usual general physical activities. Participants enrolled in the PHETHAS-1 study used the enrollment as part of their motivation for doing the exercises.

Both pain and the absence of pain were identified as barriers for doing home-based rehabilitation exercise. Pain could cause insecurity about possible medical complications, while the absence of pain could lead to the rehabilitation exercise being perceived as pointless.

**Conclusions:** The overall goal for the THA patients was to return to their habitual everyday life. This goal served as a facilitator for undertaking home-based rehabilitation exercise. Being able to perform usual activities paradoxically became a barrier for some participants, as they were more motivated to engage in general physical activity than the rehabilitation exercise.

## Introduction

In Sub-Saharan Africa, particularly in Ethiopia, where alternative modes of transportation are scarce, road transportation has emerged as a vital economic pillar. According to Asegie (2019), road transportation not only facilitates the movement of agricultural and industrial commodities but also plays a crucial role in inducing commerce, trade, and traditions. This mode of transportation has significantly contributed to the growth of feelings, cultures, and the socio-economic lives of the people in Ethiopia. By enabling the seamless transfer of goods and services from one region to another and even reaching remote villages, road transportation has been instrumental in fostering economic development and enhancing connectivity within the country.

In developing countries like Ethiopia, where alternative contemporary forms of transportation are deemed too costly, road transportation plays a crucial role in creating a network that interconnects various infrastructures. Worku (2011) highlights the significance of road transport in such nations, emphasizing its vital role due to the prohibitive costs of other transportation modes. Gebrechristos (2014) further underscores the foundational importance of road transportation in poverty alleviation efforts, providing essential urban-to-urban and urban-to-rural connectivity within the country. The acknowledgment of the value of rural road infrastructure is evident not only in Ethiopia’s overarching development strategies but also in numerous sector-specific initiatives as emphasized by Emmenegger (2012).

Traffic accidents, a significant global issue causing fatalities and property loss, are primarily attributed to human factors, vehicle conditions, and road circumstances ( Rolison et al., 2018). Research by Tiruneh et al. (2014) at Addis Ababa Black Lion Hospital revealed a correlation between patients seeking treatment and involvement in road accidents, particularly due to drunk driving. A study conducted at the University of Gondar Comprehensive Teaching and Referral Hospital highlighted a notable incidence of traffic accidents among traumatized patients, emphasizing the urgent need for preventive measures ( Honelgn & Wuletaw, 2020).

A community-based cross-sectional research study conducted in Chuko Town with a total sample size of 422 people highlighted poor road conditions as the primary cause of traffic accidents, followed by motorbike over speeding ( Tegegne, 2020). Additionally, Shamenna et al. (2021) analyzed the spatial distribution of road traffic accidents in Hawassa City, identifying sloppy driving, failure to prioritize pedestrians’ safety, driving at excessive speeds, and drivers not maintaining adequate space between vehicles as the leading factors contributing to traffic accidents in the area.

In Finoteselam town, Tadege (2020) conducted a study to determine the rate of traffic accidents, identifying drivers’ lack of driving expertise, inadequate educational level, and age of the driver, weekend time driving, and driving in the summer season as significant causes of fatal traffic accidents. Meanwhile, Tulu et al. (2013) analyzed six years of data on police-reported road traffic accidents in Ethiopia, noting a higher proportion of pedestrian involvement in collisions occurring during daylight and predominantly affecting men at the center of roads. Factors such as rollover on a road tangent, failure to follow pedestrian priority, and over speeding were highlighted as contributors to these accidents.

In Ethiopia, road traffic fatalities are considered a man-made calamity, with Hordofa et al. (2018) highlighting this issue. The Ethiopian National Road Safety Office reports a fatality rate of 114 per 10,000 cars annually, but the actual number could be higher due to an inefficient reporting system. Beyond the loss of life, traffic accidents result in significant financial burdens from medical expenses, physical suffering, permanent disabilities, and travel-related stress. These incidents also impact the household income of affected individuals, reducing their quality of life and exerting strain on the national economy.

Road traffic accidents in Ethiopia are on the rise due to unregulated rapid motorization, population growth, and road network expansion coupled with poor safety attitudes among road users. Despite limited efforts to address the issue, they fall short given the escalating problem. The lack of comprehensive research hampers effective road safety management, emphasizing the critical need for a robust accident database to pinpoint causes and drive enhancements. Without a reliable data recording system, meaningful road safety initiatives in Ethiopia, particularly in the specified region, would be severely hindered.

Road accidents and traffic management are critical concerns for road authorities, with the detection of risk factors playing a key role in enhancing road efficiency, safety, and comfort. This particular study delves into issues surrounding road surface conditions, traffic facilities, and the behavior of road users that contribute to traffic accidents. Specifically focusing on roads connecting Gondar town to Wereda town within North Gondar administrative boundaries, the research conducted a comprehensive field survey to assess current traffic accident conditions along these selected road networks. Through visual and technical inspections, the analysis identified previous accidents, road surface defects, and inadequate traffic control facilities directly impacting road safety. By pinpointing the root causes of problems within these specific road networks, the study aims to provide effective recommendations for improved safety management.

The National Road Safety Strategy for Ethiopia focuses on several key strategies to enhance road safety in the country. These strategies include strengthening the capacity of road policing leadership and operations to enforce laws related to risk factors, identifying and addressing road-related issues contributing to crashes involving various vehicle types, improving road users’ awareness of safety through platforms like social media, educational programs, and communication guidelines. Additionally, the strategy emphasizes enhancing engagement with road safety stakeholders and communities through associations, workshops, and strategic communication interventions. It aims to ensure that all motorcycle riders correctly wear standard helmets by enforcing this measure through awareness campaigns until 100% compliance is achieved. Moreover, community-based interventions are proposed to encourage public involvement in taking personal responsibility for road safety within their respective communities. Moreover, the strategy emphasizes the importance of implementing regulations and legal instruments uniformly across all regions to ensure consistent enforcement in the road transport system. Additionally, it highlights the significance of establishing, capacitating, coordinating, and integrating institutions to proactively prevent fatal and serious injury crashes within the road transport system ( Ababa, 2022).

## Methods

An inferential research study was conducted utilizing a combination of qualitative and quantitative data to examine the determinants of road traffic accidents within the administrative boundaries of the North Gondar zone, encompassing the roads bounded in 24 werdas. The primary focus of this study was on assessing and analyzing the various factors that contribute to road traffic accidents in this specific geographical area.

A secondary study sample of 603 aggregate road traffic accidents from 2015 to 2019 reveals the magnitude and frequency of traffic accident determinants involving drivers, vehicles, roads, pedestrians, and environmental factors. This data was collected from the Central Gondar zone police offices. The information on road users, vehicles, and environmental variables was sourced from the road traffic accident catalog files at police departments, maintained as wereda-level annual reports. Additionally, road inventory data for the accidents occurring on the study-bounding stretches was acquired from the Ethiopian road authority office catalog files in Gondar branch. Subsequently, onsite observations were conducted to evaluate the aspects such as traffic flow, road conditions, presence of signage, and infrastructure quality.

The traffic report formats encompass a wide array of variables crucial for analyzing and understanding traffic accidents comprehensively. These variables include age, gender, and the role of the road user (whether they were drivers, passengers, or pedestrians) involved in the accident. Additionally, the week, day, and time of the accident play a significant role in determining patterns and trends in traffic incidents. The visibility conditions at the time of the accident, distinguishing between night and daytime occurrences, were also essential factors considered. Furthermore, aspects such as light conditions, type of roads where the accidents took place, road surfaces, weather conditions prevailing during the incident, as well as the type of motor vehicles involved and their respective service years all contribute to a detailed traffic report analysis.

## Data analysis

In this study, a comprehensive analysis was conducted on the number of injuries, damages, and deaths resulting from traffic accidents within a specified period. The analysis utilized Excel version 2010 and SPSS version 2020 to perform a normal trend analysis. Absolute and relative measures were employed to characterize the accident determinants for pedestrians, drivers, passengers, vehicles, roads, and environmental factors. By utilizing these statistical techniques, the study was able to effectively represent the relationships between different variables within the accident data categories.

## Results

### Age distribution

In the span of five years, there were a total of 91 personal injuries and 507 instances of property damage. Among these, the highest records were noted in an incident that accounted for 34 percent of personal aggregate effects and 24.85 percent of property damage. Notably, the age group between 19 and 30 years old exhibited the most significant impact, with statistics showing that this demographic contributed to 56 percent of personal injuries and 62 percent of property damage, surpassing all other age brackets.

This data is further supported by Table 1, which outlines the correlation between driver age and the types of incidents. Young drivers aged between 19 and 30 were found to be the most vulnerable group, experiencing a total of 598 accidents, representing 56.7 percent of all incidents compared to older drivers above the age of 50. The heightened risk associated with younger drivers can be attributed to their propensity for engaging in riskier behaviors due to their relative lack of driving experience.

**Table 1.** Age level, type and number of road traffic accident records.

Years	Drivers’ age	Life lost	Seriously injury	Slightly injury	Property loss	Aggregate damage	Aggregate damage (%)
2015	<18	0	0	1	1	2	0.3
	19-30	31	23	22	17	93	15.6
	31-50	12	5	4	13	34	5.7
	>50	3	1	0	0	4	0.7
	Undetermined	12	8	4	1	25	4.2
2016	<18	5	3	2	1	11	1.8
	19-30	38	12	16	16	82	13.7
	31-50	11	1	3	3	18	3.0
	>50	1	0	1	0	2	0.3
	Undetermined	23	6	2	3	34	5.7
2017	<18	4	2	0	0	6	1.0
	19-30	37	13	16	13	79	13.2
	31-50	8	3	1	3	15	2.5
	>50	0	0	0	0	0	0.0
	Undetermined	19	4	5	1	29	4.8
2018	<18	2	0	0	0	2	0.3
	19-30	28	8	8	5	49	8.2
	31-50	7	3	6	2	18	3.0
	>50	1	0	0	0	1	0.2
	Undetermined	12	3	2	1	18	3.0
2019	<18	2	0	1	1	4	0.7
	19-30	15	6	10	5	36	6.0
	31-50	11	1	0	2	14	2.3
	>50	1	1	0	0	2	0.3
	Undetermined	12	5	0	3	20	3.3

### Driver’s level of education

**Figure 1.**
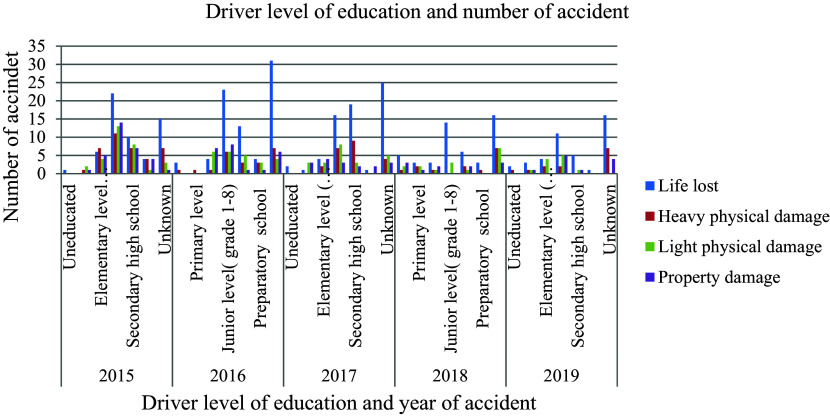
Driver level of education, type and number of road traffic accident records.

Figure 1 illustrates that, besides age, educational attainment significantly influences the occurrence of road traffic accidents. The prevailing belief is that individuals with higher levels of education exhibit greater attentiveness and a heightened concern for mitigating the risks associated with road traffic collisions compared to those with lower educational backgrounds. Consequently, a driver’s level of education equips them with the requisite knowledge, skills, and attitudes essential for ensuring vehicle safety, both while driving and as pedestrians.

In Ethiopia, the regulations for obtaining a driver’s license state that individuals can acquire a license after completing junior high school. However, concerning statistics reveal that out of 602 aggregate accidents recorded, the highest percentage of accidents, specifically 29.4 percent, involved drivers who had completed only junior high school education. This alarming statistic suggests a correlation between the occurrence of accidents and the inadequate quality of training provided to drivers who obtain their licenses through this system. Moreover, it also points towards potential issues with the approval process for driving licenses in Ethiopia, indicating that there may be flaws in the evaluation and certification of drivers under this system. The data underscores the importance of ensuring that driver training programs are comprehensive and rigorous to enhance road safety and reduce the number of accidents caused by inadequately trained drivers.

### Driver experience

**Figure 2.**
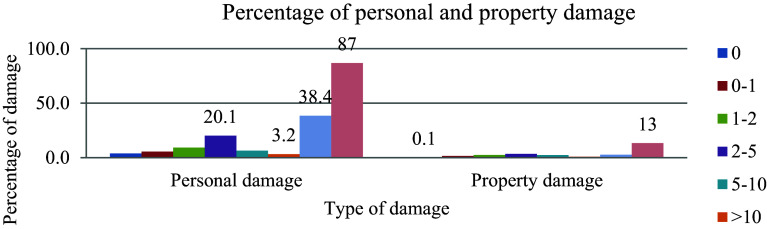
Driving experience and percentage of damages.

Driving a vehicle for an extended period and embracing the behavior of a car helps to minimize the occurrence of road traffic accidents, as illustrated in Figure 2 depicting the driving experience and percentage of damages. Unexperienced individuals are prone to flaws that can lead to mistakes in operation.

The data reveals that out of the 685 cumulative effects of aggregate damage, the highest percentage of damage, amounting to 87%, was attributed to a driver who did not possess approved licenses. This stark statistic underscores a significant issue within the control system governing driver’s licenses at traffic regulation offices, indicating a notable weakness in oversight and enforcement mechanisms.

### Drivers’ ownership responsibility

**Figure 3.**
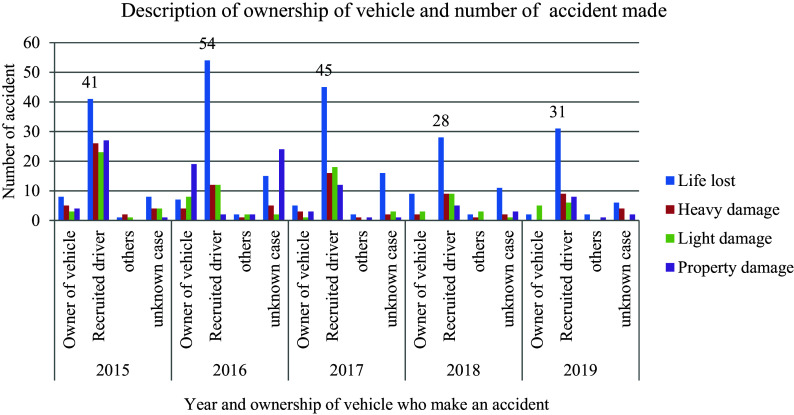
Vehicle ownership responsibility and number of damages.

In Figure 3, the data illustrates the distribution of damages and responsibilities between vehicle owners and hired drivers in road traffic accidents. Among the 622 recorded incidents, a significant majority (63.2 percent) occurred when a hired driver was behind the wheel, as opposed to the vehicle’s owner. Interestingly, when comparing the impact of employed drivers versus owners on causing accidents, it is revealed that owners contribute to an average of 14.6% of traffic collisions. This stark contrast suggests that despite owners exercising caution by limiting vehicle speed, they paradoxically increase the likelihood of being involved in a traffic collision due to various factors that may not be solely related to speed control or cautious driving practices.

### Vehicle categories

When a vehicle collides with other vehicles, a pedestrian, an animal, road curbs, existing buildings, or any immovable barrier such as a tree or electric pole, it can lead to various consequences including injury, death, and property damage.

**Figure 4.**
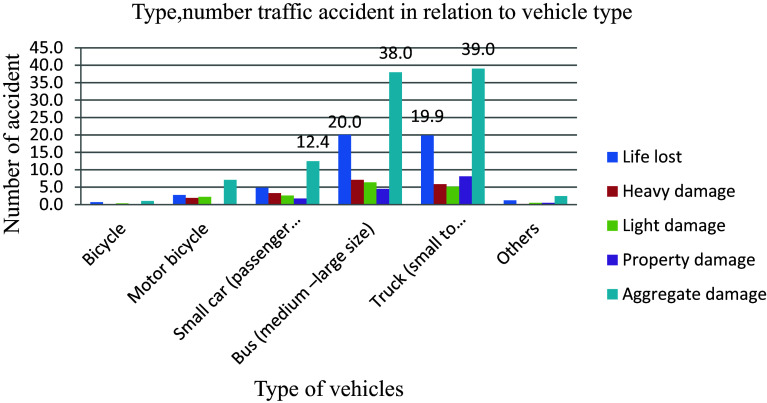
Vehicle categories and number of road traffic accident.

Trucks, as a regular sight on the country’s roadways, are a vital element of the economy, transporting commodities from one location to another to fulfil the demands of the country’s rapidly rising population. However, these large vehicles can pose a high risk to other small vehicles, especially when truck drivers are careless, leading to some of the worst roadside destruction caused by truck accidents. Trucks (small to articulated) and buses (medium to large size) are involved in the highest (39 and 38) percent of the 579 total road traffic accidents shown in Figure 4 due to their heavier and larger nature compared to passenger vehicles, increasing the risk of traffic collisions.

### Vehicle mechanical parts failure and service age

**Figure 5.**
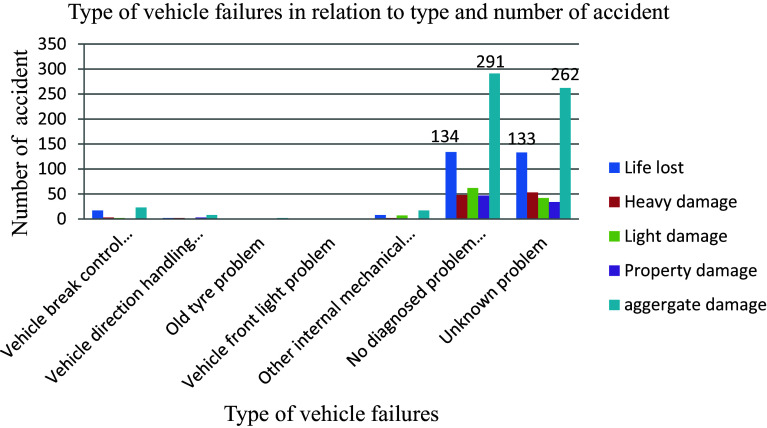
Vehicle mechanical parts failures and number of road traffic accident.

The analysis of 603 road traffic accidents revealed that a significant portion (48.3%) was attributed to diagnosed mechanical failures within vehicles rather than other internal or supportive components, as illustrated in Figure 5. These mechanical issues encompass a range of problems, including tire-related issues like blowouts and skidding caused by worn treads, brake malfunctions such as slow response times and complete failures, steering irregularities like pulling or power steering loss, malfunctioning seatbelts, faulty brakes, airbags causing injuries, defective tires, and flawed accelerators that can be unintentionally activated. The prevalence of these mechanical failures underscores their substantial contribution to road accidents, highlighting the critical importance of ensuring the proper maintenance and functioning of vehicle components to enhance road safety and mitigate the risk of accidents.

**Figure 6.**
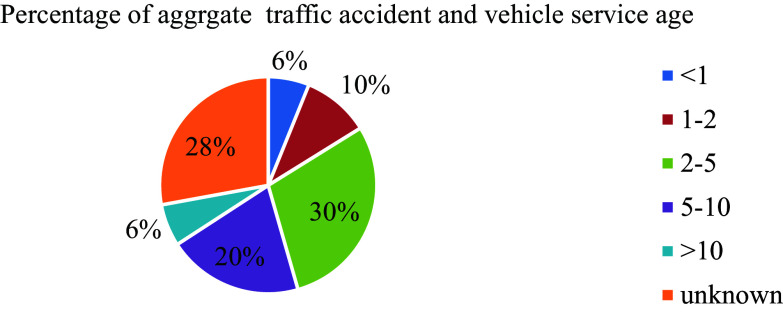
Percentage of aggregate damage and vehicle service age.

In Ethiopia, the vehicle service age plays a significant role in road traffic accidents. Despite the increasing population of motorized vehicles over time, there is a lack of approved regulations specifying vehicle service age limits, similar to the experiences of developed countries. Among 588 total recorded road traffic accidents, the highest percentages (29.4% and 27.9%) were attributed to vehicles with service years ranging from 2 to 5 years and unknown registered age groups, respectively, surpassing other service age categories as depicted in Figure 6. This trend can be attributed to the absence of a well-defined database detailing vehicle service ages within the country, leading to challenges in monitoring and regulating older vehicles on the roads.

### Road surfacing material, functional class and location

The road inventory information for this study analysis, as shown in Table 2, encompasses a detailed breakdown of various key parameters essential for the analysis. This includes the names of road segments, the length of the stretches in kilometers, Ethiopian Road Authority road identification references, road class specifications, annual average daily traffic (AADT) figures, surfacing materials utilized, and design code standards specific to the selected analysis roads. Notably, the roads under scrutiny feature asphalt-covered surfacing materials and adhere to DC-4 to DC-6 design code standards. These roads exhibit an average annual AADT ranging from 84 to 1533, reflecting varying levels of traffic volume and infrastructure requirements crucial for comprehensive analysis and planning purposes.

**Table 2.** Road inventory information of the selected analysis roads in central Gondar zone.

No	Route/Road segment	Stretch km	Road No	Road class	AADT	Surface type	Design standard
1	Bahirdar-Gondar						
1.1	Woreta-Maksegnit	74	A3-9	Trunk	1533	Asphalt	DC-6
1.2	MaksegnitAzezo	28	A3-9		1533	Asphalt	DC-6
1.3	Airport- Gondar	13	A3-10		591	Asphalt	DC-5
1.4	Gondar by pass	13					DC-6
2	Gondar Humera	290		Main Access	475	Asphalt	
2.1	Gondar Jun-Museibameb	40	C35-1		475	Asphalt	DC-5
2.2	Museibameb -Bebew River	58	C35-1		475	Asphalt	DC-5
2.3	Bebew River -Angereb River	22	C35-1		475	Asphalt	DC-5
2.4	Angereb River -Dansha	45	C35-1		475	Asphalt	DC-5
2.5	Dansha - Humra	80	C35-1		475	Asphalt	DC-5
2.6	Rawian-Lugdi	45					DC-5
3	Azezo Junction-Metma	185		Main Access	1068	Asphalt	
3.1	Azezo Junction- Bohona	35	C34-1		1068	Asphalt	DC-5
3.2	Bohona- Negadiebahir	65	C34-1		1068	Asphalt	DC-5
3.3	Negadiebahir- Shehdi	45	C34-1		1068	Asphalt	DC-5
3.4	Shehdi-Metma	40	C34-1		1068	Asphalt	DC-5
4	Shehdi- Shawera- Seraba	462		Feeder		Gravel	
4.1	ShehdiGelgo	125	E304-1		371	Gravel	DC-4
4.2	Gelgo-Shawera	157			284	Gravel	DC-4
4.3	Serab-Delgi-Shawra	100	E303-1		84	Gravel	DC-4
4.4	Airport-Gorgora	52	E33-1		192	SD	DC-4
4.5	Chuhait-Delgi	28			170	Gravel	DC-3
5	Gondar -Buya River	199		Link			
5.1	Gondar-A/Giorgise	40	B30-2		512	Asphalt	DC-6
5.2	A/Giorgise-Debark	60	B30-2		462	Asphalt	DC-6
5.3	Debark -Dagusit	24	B30-2		312	Gravel	DC-4
5.4	Dagusit-Unzo	35	B30-2		312	Gravel	DC-5
5.5	Unzo-Adirkay	20	B30-2		312	Asphalt	DC-5

**Figure 7.**
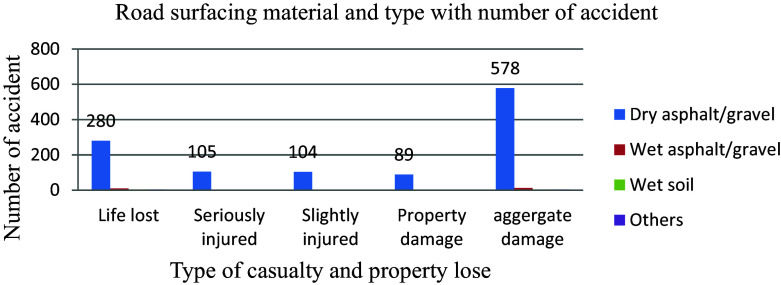
Aggregate damage and road surfacing material.

Pavement degradation and faults lead to various road hazards such as sliding, driving off tracks, inappropriate maneuvering to avoid imperfections, and increased braking distance for drivers, necessitating prompt attention from road authorities. Factors like surfacing materials, dry or wet road conditions, poor surfacing quality, and macro and micro-texture issues can result in hydroplaning, inconsistent tire-pavement contact, reduced tire grip on the road, ultimately contributing to accidents. A study revealed that a significant majority of 96.2 percent out of 601 aggregate road traffic accidents in a year occurred on dry asphalt/gravel surfaces rather than wet soil conditions. The data indicates that the occurrence of road traffic collisions is more closely associated with human factors than the condition of the road itself ( Figure 7).

**Figure 8.**
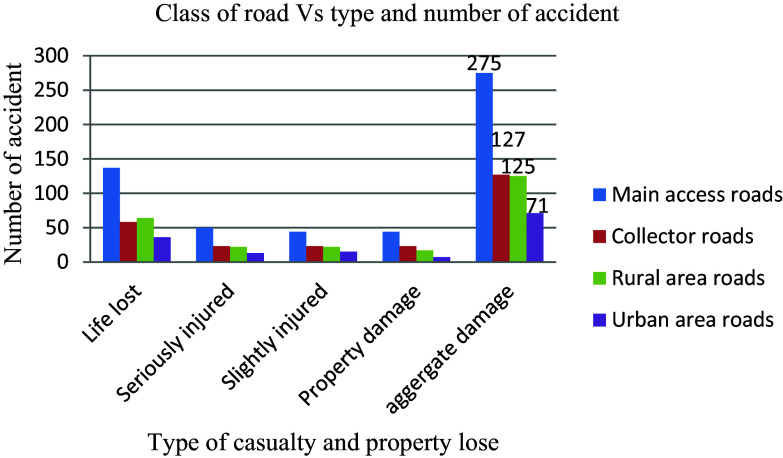
Aggregate damage and road functional classes.

Traffic safety is influenced by the different types of roadways, and while it is challenging to completely eliminate traffic collisions, mitigating their severity is achievable through enhancing road infrastructure, vehicle safety features, and promoting cautious driving behaviors. Understanding the impact of various functional classifications of roads plays a crucial role in reducing risks associated with traffic incidents. Notably, an analysis of 598 documented casualties and property losses revealed that main access roads with an annual average daily traffic (AADT) ranging from 475 to 1068 had the highest number of incidents at 275 (46%), surpassing other functional classes significantly, as illustrated in Figure 8. This underscores the detrimental effect of surpassing a route’s capacity in terms of daily traffic volume on the occurrence of road traffic accidents.

**Figure 9.**
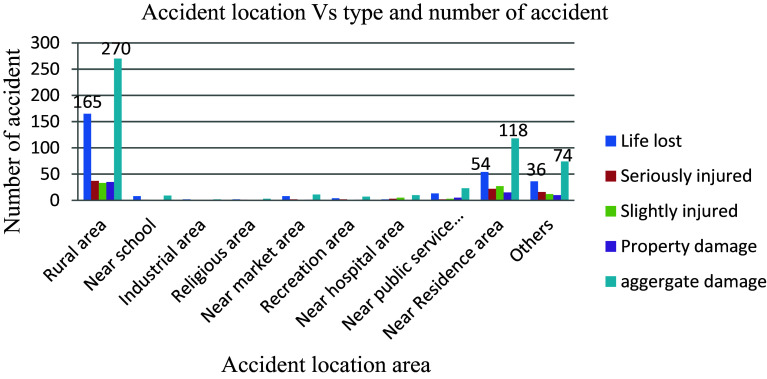
Aggregate damage and accident prone location.

The adverse impact of location and road stretch on road traffic accidents is evident in the data presented in Figure 9, where out of a total of 527 recorded accidents, 51.2% (270 accidents) occurred in rural areas and 22.4% (118 accidents) in residential areas, highlighting these as high-risk zones compared to other areas. The prevalence of accidents in rural regions suggests that local residents, particularly farmers, lack the necessary awareness and vigilance to safeguard themselves against road traffic incidents. Conversely, the concentration of young children near schools poses another significant risk factor as they often exhibit limited understanding of crucial aspects such as vehicle speeds, proper sidewalk usage, recognizing pedestrian crosswalks, and other behaviors essential for reducing the likelihood of road traffic accidents.

### Weather condition and day time situation

Weather significantly impacts driving capabilities and vehicle performance through visual impairments, precipitation, severe winds, and temperature extremes. These factors directly affect traction, stability, and maneuverability of vehicles on the road. According to Table 3 data, the majority of weather-related crashes, accounting for 91.9 percent (547 incidents), occur during visible daylight conditions, with 91.2 percent (539 cases) happening under such lighting circumstances. This highlights the critical role that weather conditions play in road safety and underscores the need for drivers to adapt their driving behavior accordingly to mitigate risks associated with adverse weather conditions.

**Table 3.** Aggregate damage and weather condition.

Weather condition	Life lost	Seriously injured	Slightly injured	Property damage	aggregate damage	Aggregate damage (%)
Normal weather condition	275	86	95	83	539	91.2
Foggy	1	0	0	1	2	0.3
Cloudy	0	1	0	2	3	0.5
Light raining	1	4	1	2	8	1.4
Water flood (heavy rain)	1	0	0	0	1	0.2
Windstorm	0	0	0	0	0	0.0
Dust sky coverage	2	1	1	0	4	0.7
Warm climate	13	8	7	3	31	5.2
Bone chilling coldness	1	0	0	0	1	0.2
Others	1	1	0	0	2	0.3

### Time and day wise distribution

**Figure 10.**
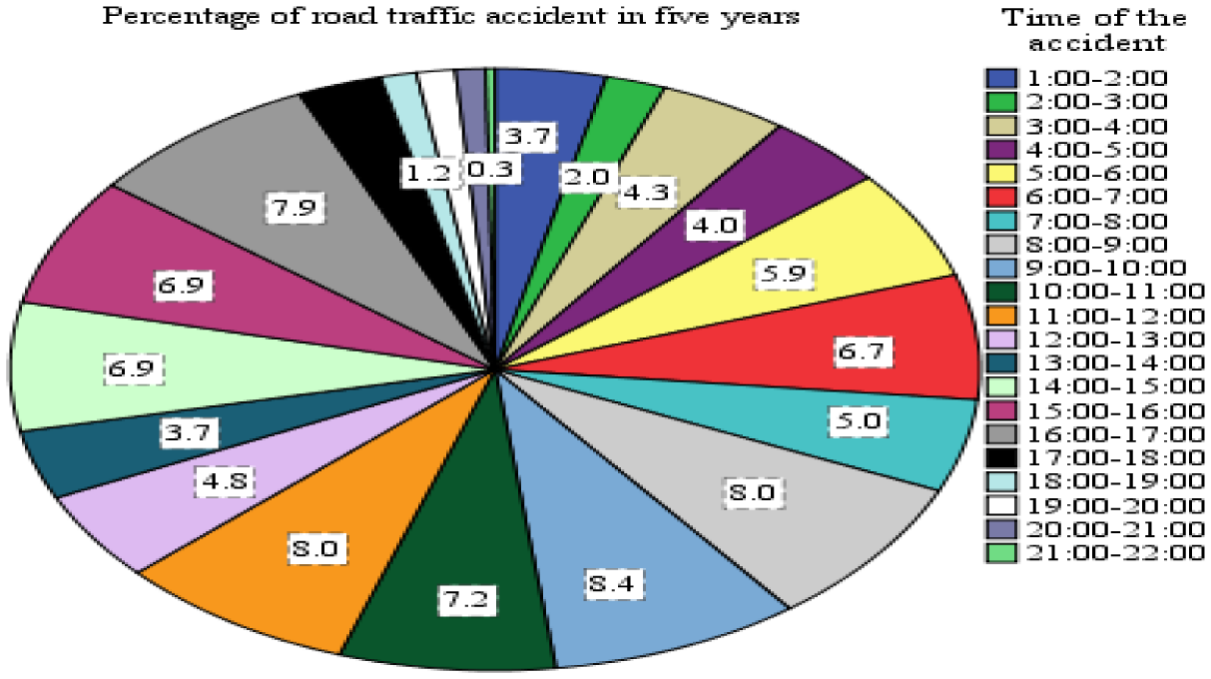
Percentage of traffic accident and time wise distributions.

In the study area, Figure 10 illustrates the time-wise distribution of road accidents, showcasing a significant variance in road traffic accidents across different times of the day. Among the 598 total recorded aggregate accidents, the highest percentages were observed during two specific time intervals: 8:00 to 12:00 and 15:00 to 17:00, accounting for 31.6% and 22% of the accidents, respectively. This data indicates that a substantial portion of traffic accidents occurred during daytime hours rather than at night, suggesting a correlation between higher traffic flow and increased accident rates during daylight hours.

**Figure 11.**
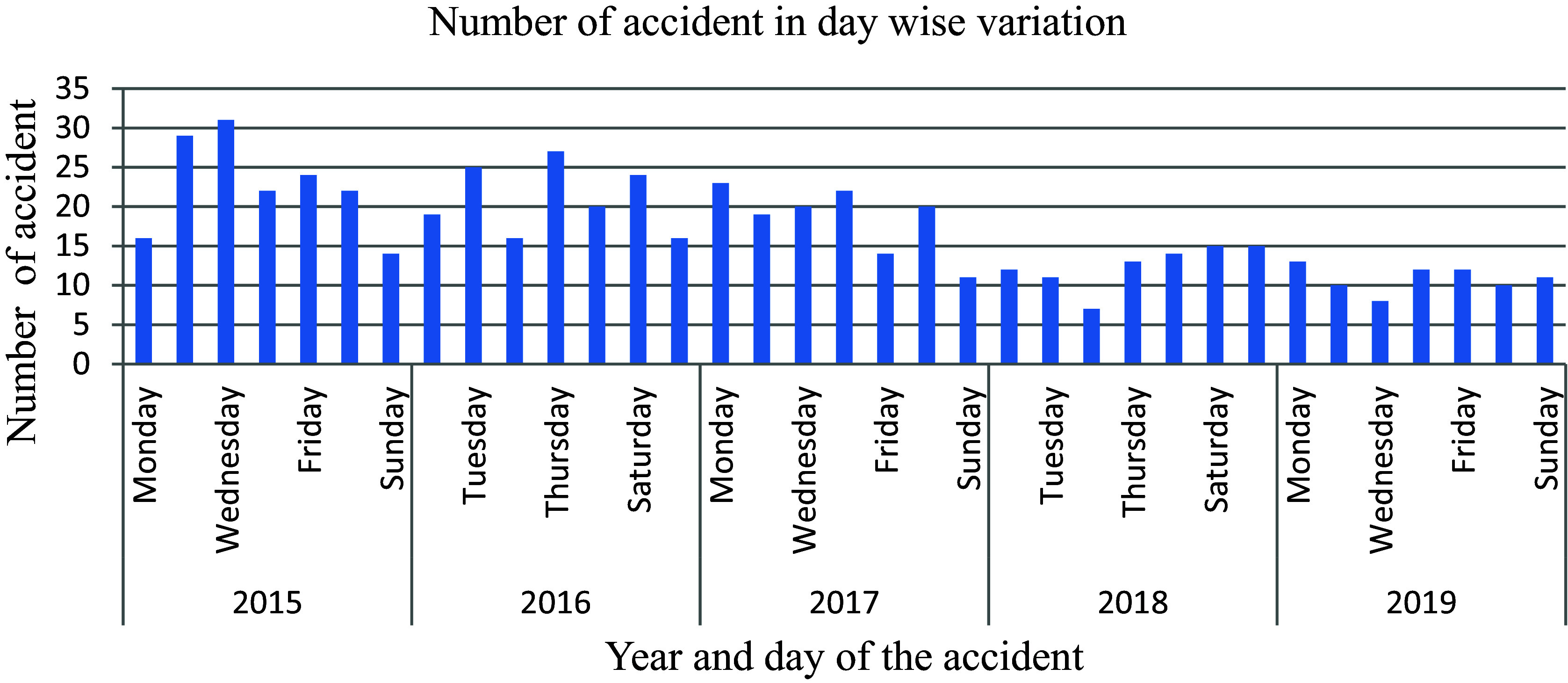
Number of road traffic accidents in day-wise variations.

The analysis presented in Figure 11 illustrates that there was a mere 2% fluctuation in the number of traffic accidents reported from Monday to Saturday, contrasting significantly with the higher variability observed on Sundays among the total of 597 incidents recorded by the police department. Subsequent to a specific date, there has been an 11.2% decrease in the documentation of road traffic accidents, indicating a reduction in both commercial vehicle activity and passenger mobility on Sundays, consequently leading to a decline in the occurrence of accidents.

### Category of casualty

**Figure 12.**
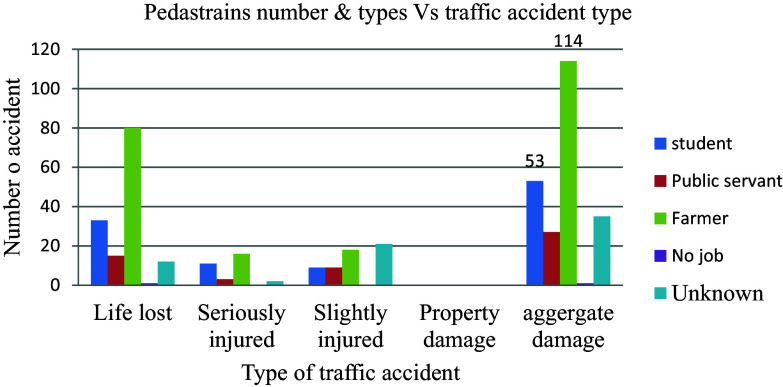
Aggregate damage with the type of casualty.

The majority of the total of 230 aggregate damages that occurred, amounting to 89 (38.7%) incidents of road traffic injuries, affected farmers the most with 114 victims (49.6%). Students were the next most impacted group, constituting 53 victims (23%) more than any other category of people, as shown in Figure 12.

## Discussions

In Ethiopia, including the study area, the basic accident data is solely collected by the police and recorded in a notebook using a locally developed standard format. Typically, this data collection occurs when the police attend a road accident; however, there are instances where accidents are reported post-incident at a police station. This delay in reporting can result in crucial information regarding the causative factors of traffic accidents being omitted, leading to potential misinterpretation of recommended actions to address road safety issues. Therefore, it is imperative that Ethiopia establishes formal, centrally developed traffic accident recording standard formats that can be uniformly adopted across the country to ensure consistent and accurate registration of accident data.

In place of manual recording traditions, there should be an automated computerized web-based database recording system that works through the country regions also applicable, which will enable the traffic police offices and road safety stakeholders to get valuable online traffic accident information at various levels easily and quickly. This can be done cooperatively with the federal police commission’s traffic accident analysis department and global consulting agencies that have good exposure for traffic database management systems. The system may include different subsystems for administrative and accident registration to meet its core functionalities, which are managed in different local languages.

Despite the significant impact of traffic accidents, the cooperation between the road transport, traffic police departments, and Ethiopian road authority and infrastructure development offices is inadequate. To address this issue effectively, a uniform rule should be issued by the Amhara regional bureau to ensure consistent coordination among these sectors for efficient traffic accident management.

In the study area, young drivers are obtaining driver’s licenses without taking the necessary trainings and skills for driving safely. One of the primary causes of these mishaps is the presence of younger drivers with a junior level of education, inexperience, and a lower sense of ownership responsibility. Studies have shown that drivers under the age of 25 are more likely to be involved in traffic accidents compared to seniors, more experienced drivers globally. This is principally due to their inability to assess risks accurately and their propensity for unsafe driving practices, such as over speeding, aggressive motoring, and driving while preoccupied.

In many parts of the developing countries, the level of education and experience in young drivers also plays a role in traffic accidents. A lack of decent education or gaining relevant experience often correlates with less understanding of traffic rules and regulations. These young drivers may not have access to proper driver’s education programs, which may result in poor driving habits and an increased likelihood of accidents. This situation is exacerbated by ineffective enforcement of traffic laws, leading young drivers to not take them seriously, and further worsening the situation. Additionally, youthful hired drivers may not feel the same level of responsibility as the owners for the maintenance and proper functioning of their vehicles, contributing to a higher rate of accidents.

In the area, the contribution of heavier loading capacities and the service age of trucks to traffic accidents is more significant compared to others due to several factors. Firstly, in the area including most developing countries, there are often lax regulations and enforcement regarding vehicle weight limits, leading to overloaded trucks on the roads. These overloaded trucks pose a higher risk as they are more challenging to maneuver and control, especially on poorly maintained road infrastructure. Additionally, the aging trucks prevalent in these area are more prone to mechanical failures and breakdowns, increasing the likelihood of accidents. Moreover, inadequate maintenance practices and limited access to quality spare parts further exacerbate the safety risks associated with older trucks operating in these country settings. The combination of these factors creates a hazardous environment where the probability of accidents involving trucks with heavier loads and older service ages is significantly elevated, highlighting the urgent need for improved regulations, enforcement, and investment in modernizing truck fleets to enhance road safety in developing countries.

In the study area, the primary causes of accidents are predominantly attributed to driver behavior-related factors rather than other causative elements. **Bad driving behaviors**, such as tailgating (driving too closely behind another car), exceeding the design speed, using a phone while driving, failing to indicate when turning directions, dangerous overtaking, excessive braking, a lack of understanding of roundabouts, loading and seating above the specified capacities and number of chairs, illegally parking and loading out of public stations, are identified as significant contributors to road accidents in these regions.

Centrally in regional or zone administrative areas, traffic accident safety trainings should be conducted at least once a year for the relevant traffic accident role players. These trainings should be supported by cloud computerized systems that control and monitor the recording systems to ensure effectiveness.

To reduce the impacts of traffic accidents in the area, traffic police officers should take on higher responsibility. A discouraging system needs to be extended and networked across the zones to control illegal communications between the commercial vehicle drivers and police officers at road checkpoints. Priority should be given to passenger safety and concern for human life lost by the police officers. This can be easily managed through annual scheduled panel discussions, evaluations and reports. Setting a good example can be achieved by providing enhanced rewards and incentives to individuals who perform better, thereby encouraging them to excel.

In the present day, various initiatives such as annual traffic accident day celebrations, the establishment and training of student traffic police clubs, the deployment of student police controls near school gates, and encouraging pedestrians to walk on the left side of the road are being actively practiced by members of the public, students, and local communities. These efforts are crucial in promoting awareness and safety regarding traffic accidents and should be further encouraged and sustained. It is imperative that all stakeholders including individuals, private sector entities, government bodies, and non-governmental organizations collaborate effectively to collectively work towards achieving a goal of zero percent road traffic accidents through continuous education, enforcement, and community engagement.

## Conclusions

Ethiopia, including the study area, is currently facing a significant road safety crisis, with thousands of individuals losing their lives annually due to road-related incidents. Despite the government’s efforts to mitigate these issues, the prevalence of road traffic accidents continues to rise. To delve deeper into this pressing matter, a descriptive research approach was employed, utilizing both qualitative and quantitative data sourced from secondary sources. The data collection process involved gathering information from previous traffic police reports spanning five years (2015–2019) in the North Gondar zone police departments. Additionally, functional road data was obtained from the Ethiopian Road Authority offices located in Gondar branch. This meticulous data collection strategy aimed to provide a comprehensive understanding of the road safety challenges faced in the region and lay the groundwork for potential interventions and solutions.

The study focuses on analyzing the frequency of traffic accidents caused by driver-related factors, including age, sex, level of education, driving experience, ownership responsibility of a vehicle and driver responsibility. It also examines the time and day-wise distribution of traffic accidents, vehicle-related factors such as failures of mechanical parts of the vehicle and its service age, road geometric design, and construction considerations like road inventory information, road surfacing materials, pedestrians, and environmental factors.

## Data availability

Zenodo. Magnitude and determinants of road traffic accidents in North Gondar Zone, Amhara Region, Ethiopia. DOI: https://doi.org/10.5281/zenodo.6948626 ( Molla, 2022).

This project contains the following underlying data:

- Number and types of a road traffic accidents in relation to road and road user, environmental and time related and vehicle related factors

Data are available under the terms of the Creative Commons Zero “No rights reserved” data waiver (CC BY 4.0 Public domain dedication).
