# Supplementary material for: Magnitude and determinants of road traffic accidents in North Gondar Zone; Amhara Region, Ethiopia
Source: F1000Res. 2024 Jul 31;12:367. Originally published 2023 Apr 5. [Version 3] doi: 10.12688/f1000research.123111.3 (PMC12504942; doi:10.12688/f1000research.123111.3)
Supplement: Peer review history

# Reviewer report: Reviewer response for version 3

**DOI:** 10.5256/f1000research.169738.r413378

**Published:** 2025-10-07

**Authors:** Paulo Infante

**Affiliations:** 1 Universidade de Évora, Évora, Portugal

The manuscript analyses five years (2015–2019) of police-reported road traffic accidents in the North Gondar Zone, Ethiopia. It aims to identify the determinants and patterns of road traffic crashes by combining qualitative and quantitative data. The study describes accident frequencies according to driver characteristics (age, education, driving licence, experience, ownership), vehicle type and age, road class and surface, location (urban/rural), weather, time of day, and day of week.

 The authors highlight that most accidents involved young drivers (≈19–30 years), unlicensed or inexperienced drivers, heavy vehicles (trucks/buses), rural roads, and daytime conditions. The paper concludes that driver behaviour, law enforcement, and database improvements are key to reducing accidents.

 The overall narrative is understandable, but some terms are used inconsistently (e.g., “life lost”, “aggregate damage”). The literature review is short, with several outdated or local references. The link between the objectives, literature, and results is not always clear.

 The study claims to combine qualitative and quantitative approaches, yet only descriptive tabulations from police records are shown. No qualitative data, sampling, or analytical methods are described. The design is therefore only partly appropriate for the stated goal of identifying “determinants.”

 Although data sources and variables are listed, the manuscript lacks crucial details: inclusion/exclusion criteria, data cleaning, coding, handling of missing data, and analytical procedures. Without these, full replication is not possible.

 The analysis is purely descriptive (counts and percentages). Inferential statements about determinants are made without supporting statistical models or confidence intervals. Terms such as “significant” and “determinant” are used loosely.

 The authors state that the aggregated police dataset is publicly available in Zenodo. However, codebooks and analysis scripts should also be included to enhance reproducibility.

 The conclusions (e.g., “driver behaviour is the main determinant”) are plausible but not quantitatively demonstrated. They should be explicitly linked to the evidence presented, avoiding causal wording when only descriptive results are available.

**1. Clarity and structure**

- The manuscript would benefit from clearer alignment between **objectives, methods, results, and conclusions**.
- The stated goal of identifying *determinants* suggests an analytical design, but the current version remains descriptive.
- The writing is generally clear but should be revised for conciseness and consistency of terminology (e.g., “fatalities” instead of “life lost”; “property damage only” instead of “aggregate damage”).
- The abstract and main text should be rewritten to accurately reflect what was done and found.

**2. Study design and data description**

- Specify the **type of study** (e.g., *retrospective descriptive analysis of police-recorded crashes*).
- Provide details on the **data source**: institution, coverage, completeness, and any permissions obtained.
- Clarify whether any **qualitative component** was performed; if not, remove all mentions of it. If qualitative data were collected, describe sampling, instruments, and analytical approach (e.g., thematic analysis).

**3. Statistical analysis**

- The paper currently presents descriptive summaries only. To justify the use of the term *determinants*, include appropriate inferential analyses, for example:
  - **Chi-square tests** for associations between crash severity and categorical variables.
  - **Logistic or ordinal regression** for severity outcomes (fatal vs non-fatal, etc.), adjusting for confounders such as age, licence, road class, and vehicle type.
  - **Poisson or negative binomial models** for accident counts, using exposure variables such as traffic volume or road length (log-offset).
- Report **confidence intervals** and p-values and interpret them cautiously.
- Avoid causal wording unless the design allows it.

**4. Presentation of results**

- Replace absolute percentages with **rates** where possible (e.g., crashes per 10⁵ population, per 100 million vehicle-km, or per km of road).
- Clearly label figures and tables: specify denominators, categories, and note how unknown values were handled.
- Verify that all percentages add up correctly within analytical frames.
- Consider visual summaries (e.g., bar charts by driver age group or heatmaps by road type and time of day) to improve readability.

**5. Discussion and literature**

- Expand the discussion with **recent and international studies** on road traffic injury determinants (e.g., driver demographics, road environment, enforcement).
- Compare your findings directly with those studies, indicating convergences or differences and possible explanations (exposure, infrastructure, enforcement).
- Include a concise **limitations subsection**, addressing:
  - Under-reporting and selection bias in police data;
  - Lack of exposure measures;
  - Missing variables such as alcohol use, speed, or distraction;
  - Aggregated nature of data (ecological limitations).

**6. Conclusions**

- Re-write to reflect the actual evidence.
- Rather than asserting determinants, state that the study *identified descriptive patterns suggesting higher crash frequencies among young or unlicensed drivers, heavy vehicles, and rural roads*.
- Separate **policy recommendations** (e.g., driver training, data systems) from the summary of results.
- Avoid over generalization beyond the dataset’s scope.

This manuscript addresses an important and under-studied topic in Ethiopian road safety. The data coverage is valuable, and the descriptive results are informative for policy. However, as currently written, the paper falls short of the analytical rigor implied by its title and objectives.

Is the work clearly and accurately presented and does it cite the current literature?

Partly

If applicable, is the statistical analysis and its interpretation appropriate?

Partly

Are all the source data underlying the results available to ensure full reproducibility?

Yes

Is the study design appropriate and is the work technically sound?

Partly

Are the conclusions drawn adequately supported by the results?

Partly

Are sufficient details of methods and analysis provided to allow replication by others?

No

Reviewer Expertise:

Statistical Modelling and Data Analysis

I confirm that I have read this submission and believe that I have an appropriate level of expertise to confirm that it is of an acceptable scientific standard, however I have significant reservations, as outlined above.

# Reviewer report: Reviewer response for version 3

**DOI:** 10.5256/f1000research.169738.r309348

**Published:** 2024-08-22

**Authors:** Aliyu Mustapha

**Affiliations:** 1 Industrial and Technology Education Department, Federal University of Technology Minna, Minna, Niger, Nigeria; 2 Mechanical Engineering, Universiti Teknologi PETRONAS, Bandar Seri Iskandar, Perak, Malaysia

Supporting or contradicting the discussions with citations is crucial for adding credibility and depth to the arguments presented.

 - Unbold " **Bad driving behaviors**" at the discussion sections

 - 7 out of 10 paragraphs in the discussion section, started with the word "In". Kindly substitute with other suitable words.

Is the work clearly and accurately presented and does it cite the current literature?

Partly

If applicable, is the statistical analysis and its interpretation appropriate?

Yes

Are all the source data underlying the results available to ensure full reproducibility?

Partly

Is the study design appropriate and is the work technically sound?

Yes

Are the conclusions drawn adequately supported by the results?

Partly

Are sufficient details of methods and analysis provided to allow replication by others?

Partly

Reviewer Expertise:

Road Traffic Accidents, Automobile Technology, Technical Vocational Education and Training, Educational Technology, ICT in Education, ICT for Education, Road Signs and Ergonomic

I confirm that I have read this submission and believe that I have an appropriate level of expertise to confirm that it is of an acceptable scientific standard.

# Response

**Published:** 2024-08-23

**Authors:** Abay Semegnew Molla

**Affiliations:** University of Gondar, Ethiopia

Thank you very much for giving me valuable lessons and comments.

# Reviewer report: Reviewer response for version 3

**DOI:** 10.5256/f1000research.169738.r309350

**Published:** 2024-08-07

**Authors:** Svetlana Cociu

**Affiliations:** 1 Department of Preventive Medicine, Nicolae Testemitanu State University of Medicine and Pharmacy, Chisinau, Moldova

I wrote my comments previously, but there is still needed revision from the authors. My decision is approved with revision.

Is the work clearly and accurately presented and does it cite the current literature?

Yes

If applicable, is the statistical analysis and its interpretation appropriate?

Partly

Are all the source data underlying the results available to ensure full reproducibility?

Partly

Is the study design appropriate and is the work technically sound?

Yes

Are the conclusions drawn adequately supported by the results?

No

Are sufficient details of methods and analysis provided to allow replication by others?

Partly

Reviewer Expertise:

Health promotion, injury prevention, road injury prevention, adolescents health

I confirm that I have read this submission and believe that I have an appropriate level of expertise to confirm that it is of an acceptable scientific standard, however I have significant reservations, as outlined above.

# Reviewer report: Reviewer response for version 2

**DOI:** 10.5256/f1000research.158773.r282418

**Published:** 2024-06-17

**Authors:** Puspa Raj Pant

**Affiliations:** 1 Social Sciences, Nexus Institute of Research and Innovation (NIRI), Kathmandu, Nepal; 2 College of Health Science and Society, University of the West of England, Bristol, England, UK

Dear Author and Editor

 I wonder the Abstract of this version is completely different to what the study is about. It must be an error or negligence. Please correct this. 

 Also, the revised manuscript does not address my previous comments. Since the manuscript remains almost the same as previous version, my decision would also be the same.

Is the work clearly and accurately presented and does it cite the current literature?

Partly

If applicable, is the statistical analysis and its interpretation appropriate?

No

Are all the source data underlying the results available to ensure full reproducibility?

Yes

Is the study design appropriate and is the work technically sound?

No

Are the conclusions drawn adequately supported by the results?

No

Are sufficient details of methods and analysis provided to allow replication by others?

No

Reviewer Expertise:

Road Traffic Injury Prevention; Injury Prevention

I confirm that I have read this submission and believe that I have an appropriate level of expertise to state that I do not consider it to be of an acceptable scientific standard, for reasons outlined above.

# Reviewer report: Reviewer response for version 2

**DOI:** 10.5256/f1000research.158773.r282416

**Published:** 2024-06-05

**Authors:** Aliyu Mustapha, Abdullahi Raji Egigogo

**Affiliations:** 1 Industrial and Technology Education Department, Federal University of Technology Minna, Minna, Niger, Nigeria; 2 Mechanical Engineering, Universiti Teknologi PETRONAS, Bandar Seri Iskandar, Perak, Malaysia; 3 Software Engineering and Cyber Security, Al-Qalam University, Katsina, Katsina State, Nigeria

**Objectives and Hypotheses**

 Clearly state the objectives and hypotheses; this will guide you in drawing the conclusion and making recommendations for the study.

**Methodology**

 The Qualitative aspect of this study was not reported.

**Inferential Statistics**

- Formulate hypotheses based on the research questions.
- Techniques such as chi-square tests for categorical variables, t-tests, and ANOVA for continuous variables are used to determine the significance of different factors.
- Use regression analysis to identify the relationship between independent variables (Such as road conditions and driver behaviour) and dependent variables (such as accident severity and frequency).

**Data Analysis**

- Is there SPSS version 2020? Kindly check the versions of SPSS
- How did you analyse the qualitative data?

**Discussions**

- Re-visit the discussions and support your findings with citation(s).

**Conclusions**

 Conclude based on your research objectives. Re-write this aspect

**Recommendations**

- Provide recommendations based on your research objectives.

Is the work clearly and accurately presented and does it cite the current literature?

Partly

If applicable, is the statistical analysis and its interpretation appropriate?

Yes

Are all the source data underlying the results available to ensure full reproducibility?

Partly

Is the study design appropriate and is the work technically sound?

Yes

Are the conclusions drawn adequately supported by the results?

Partly

Are sufficient details of methods and analysis provided to allow replication by others?

Partly

Reviewer Expertise:

Road Traffic Accidents, Automobile Technology, Technical Vocational Education and Training, Educational Technology, ICT in Education, ICT for Education, Road Signs and Ergonomic

We confirm that we have read this submission and believe that we have an appropriate level of expertise to confirm that it is of an acceptable scientific standard, however we have significant reservations, as outlined above.

# Reviewer report: Reviewer response for version 1

**DOI:** 10.5256/f1000research.135182.r192796

**Published:** 2023-10-31

**Authors:** Aliyu Mustapha

**Affiliations:** 1 Industrial and Technology Education Department, Federal University of Technology Minna, Minna, Niger, Nigeria; 2 Mechanical Engineering, Universiti Teknologi PETRONAS, Bandar Seri Iskandar, Perak, Malaysia

**Strengths**

1. Relevance of the Topic: The article addresses a highly relevant and significant issue – road traffic accidents, which are a major public health concern not only in Ethiopia but globally.
2. Clear Objective: The article clearly states its objective, which is to examine the primary contributing variables to road traffic accidents in a specific region, the North Gondar Zone. This clear focus helps readers understand the study's purpose.
3. Comprehensive Data: The study uses a five-year dataset, which provides a substantial amount of data to analyze the factors contributing to road traffic accidents. This strengthens the validity of the findings.
4. Recommendations: The article provides practical recommendations for reducing road traffic accidents, specifically focusing on controlling bad driving behaviors and implementing advanced computerized traffic accident safety training and recording systems. These recommendations have the potential to impact policy and safety practices.
5. Structured Layout: The article follows a logical structure, with clear sections for the introduction, methods, results, discussion, and conclusion. This organization makes it easy for readers to follow the research's progression.
6. Use of References: The article effectively references prior research to provide context and support the need for this study.
7. Clear Recommendations: The article provides clear and practical recommendations for improving the road safety situation in Ethiopia and the study area. Suggestions for the implementation of an automated, web-based traffic accident recording system and the need for collaboration among various sectors are valuable.
8. Emphasis on Driver Behavior: The article appropriately highlights the importance of driver behavior as a significant contributor to road traffic accidents, and it provides specific examples of bad driving behaviors. This focus on behavior addresses a critical aspect of road safety.
9. Emphasis on Collaboration: The emphasis on improving cooperation between different sectors, including road transport, traffic police, and the Ethiopian road authority, is a valuable point for addressing road safety comprehensively.
10. Discussion of Ongoing Practices: The article highlights existing practices such as annual traffic accident day celebrations, student traffic police formation, and training, which should be promoted to enhance traffic accident safety. These practices are positive and should be continued.

**Weaknesses**

1. Data Collection Details: The article lacks specific details about the data collection and analysis methods used. Readers may want more information on how data was gathered, processed, and analyzed to ensure the validity and reliability of the results.
2. Literature Review Depth: While the article references existing literature, it could benefit from a more comprehensive literature review that delves deeper into global and regional trends in road traffic accidents. This would provide a broader context for the specific findings in the North Gondar Zone.
3. Limited Discussion Implications: The discussion section could be expanded to explore the broader implications of the findings. How might these findings impact road safety policies, practices, or interventions not only in the North Gondar Zone but also in other regions? Providing a more extensive discussion could enhance the article's impact.
4. Clarity in Language: While the article is generally clear, there are areas where the language could be refined for greater clarity and precision. Some sentences are complex and might benefit from simplification.
5. Lack of Data: The article does not present any new data or statistical analysis related to road traffic accidents in the North Gondar Zone. It mainly consists of recommendations and general statements about the problem without supporting data or analysis.
6. Repetitive Statements: The article repeats certain points, such as the importance of an automated recording system and the role of driver behavior, without providing substantial additional insights or examples.
7. Absence of Literature Review: The article does not reference existing literature or research on road safety in Ethiopia or the North Gondar Zone. Including a literature review would provide context for the recommendations and reinforce the importance of the issue.
8. It is not always good to start a paragraph with citation, always start a paragraph with your own words, then support with citation(s). “Check the first, fourth and fifth paragraphs of the introduction”.
9. The first-two paragraphs of the conclusion are “Summary” not “Conclusion”. Re-write the conclusion based on your objectives.
10. Data analysis used is mainly descriptive. You should add inferential statistics to make educated guesses or inferences about populations based on data from a sample. Formulate hypotheses based on your research questions.
11. Provide a subheading for recommendations and limitation(s) based on your research objectives

**Conclusion**

 In conclusion, the article addresses a crucial issue and provides valuable recommendations to mitigate road traffic accidents. Its strengths lie in its relevance, clear objectives, comprehensive data, and practical recommendations. However, there is room for improvement in terms of providing more details about data collection methods, expanding the literature review, and further discussing the broader implications of the research findings.

Is the work clearly and accurately presented and does it cite the current literature?

Partly

If applicable, is the statistical analysis and its interpretation appropriate?

Yes

Are all the source data underlying the results available to ensure full reproducibility?

Partly

Is the study design appropriate and is the work technically sound?

Yes

Are the conclusions drawn adequately supported by the results?

Partly

Are sufficient details of methods and analysis provided to allow replication by others?

Partly

Reviewer Expertise:

Road Traffic Accidents, Automobile Technology, Technical Vocational Education and Training, Educational Technology, ICT in Education, ICT for Education, Road Signs and Ergonomic

I confirm that I have read this submission and believe that I have an appropriate level of expertise to confirm that it is of an acceptable scientific standard, however I have significant reservations, as outlined above.

# Reviewer report: Reviewer response for version 1

**DOI:** 10.5256/f1000research.135182.r212327

**Published:** 2023-10-31

**Authors:** Mireia Faus

**Affiliations:** 1 University of Valencia, Valencia, Spain

The paper analyzes the main variables contributing to road accidents in a region of Ethiopia. The causes and variables related to road accidents are a widely researched topic internationally. However, the study in a specific region of Ethiopia may have important practical applications at the local level, as well as for regions or countries with similar socio-demographic characteristics.

 In the following, I will make some recommendations that I consider can improve the quality of the paper.

 I recommend further developing the introduction, including the main factors involved in road accidents, such as the human factor, the infrastructure factor and the vehicle factor. In this sense, two subsections could be included, referring to 1) Literature review, synthesizing the main research on the subject, especially those carried out in emerging countries, and 2) The case of Ethiopia, explaining the country's situation with respect to road accidents and citizens' mobility.

 The methodology should describe the variables evaluated, the type of statistical analysis carried out, the ethical aspects of the study, etc.

 The data are merely descriptive. Therefore, the discussion cannot be a mere summary of the data obtained. This section should explain and contrast the data obtained with other research in this field of study. Thus, the authors should answer questions such as: were the results in accordance with expectations, are the results congruent with other similar research? And, if not, what elements explain the discrepancies that have occurred? The limitations of the study should also be included in a specific section.

Is the work clearly and accurately presented and does it cite the current literature?

Partly

If applicable, is the statistical analysis and its interpretation appropriate?

Partly

Are all the source data underlying the results available to ensure full reproducibility?

Partly

Is the study design appropriate and is the work technically sound?

Partly

Are the conclusions drawn adequately supported by the results?

No

Are sufficient details of methods and analysis provided to allow replication by others?

No

Reviewer Expertise:

Road traffic; safety; communication; public health

I confirm that I have read this submission and believe that I have an appropriate level of expertise to state that I do not consider it to be of an acceptable scientific standard, for reasons outlined above.

# Reviewer report: Reviewer response for version 1

**DOI:** 10.5256/f1000research.135182.r192802

**Published:** 2023-10-31

**Authors:** Puspa Raj Pant

**Affiliations:** 1 Social Sciences, Nexus Institute of Research and Innovation (NIRI), Kathmandu, Nepal; 2 College of Health Science and Society, University of the West of England, Bristol, England, UK

Author could have referred to some global literature to give an idea about recognised risk factors and effective measures to address them. Some of the paragraphs in introduction section look more appropriate to be mentioned in the Discussion.

 The objective mentioned in Abstract, Introduction, and Discussion section is not consistent. The design of the primary study conducted on road surfaces in North Gondar area is not well described. Although "Qualitative" design is mentioned, no results or discussion is seen in the manuscript. 

 The Methods section needs clear details on Quantitative, Qualitative and Secondary data analysis. Currently the data analysis is very thin. The author could have generated some crosstabulations.

 The manuscript has not made justice to identify the "determinants" as mentioned in the Title. 

 The first three paragraphs of Discussion look more like recommendations. The author could compare and contrast their findings (as some examples mentioned in introduction section).

 Conclusion looks like a summary of methods.

 References need to be cited in full in the Bibliography: Tegegne KT; Honelgn A; Wuletaw T; Emmenegger R.

Is the work clearly and accurately presented and does it cite the current literature?

Partly

If applicable, is the statistical analysis and its interpretation appropriate?

No

Are all the source data underlying the results available to ensure full reproducibility?

Yes

Is the study design appropriate and is the work technically sound?

No

Are the conclusions drawn adequately supported by the results?

No

Are sufficient details of methods and analysis provided to allow replication by others?

No

Reviewer Expertise:

Road Traffic Injury Prevention; Injury Prevention

I confirm that I have read this submission and believe that I have an appropriate level of expertise to state that I do not consider it to be of an acceptable scientific standard, for reasons outlined above.

# Reviewer report: Reviewer response for version 1

**DOI:** 10.5256/f1000research.135182.r192799

**Published:** 2023-08-14

**Authors:** Svetlana Cociu

**Affiliations:** 1 Department of Preventive Medicine, Nicolae Testemitanu State University of Medicine and Pharmacy, Chisinau, Moldova

The authors reflect a very informant issue. Results are significant for the scientific environment. I would recommend a slight modification: 

 In the abstract, the result section needs to refer to the results obtained and all the variables used need to be replaced in the methodology part. The methodology is not completed, it would be interesting more details about how data were obtained and what statistical tools were used.  The result part is well organized - I suggest revising the figures and in some of them - to do average of the study period. The discussion part needs revision - data comparable with other studies should be given. The conclusion needs to refer to some solutions based on the obtained results.

Is the work clearly and accurately presented and does it cite the current literature?

Yes

If applicable, is the statistical analysis and its interpretation appropriate?

Partly

Are all the source data underlying the results available to ensure full reproducibility?

Partly

Is the study design appropriate and is the work technically sound?

Yes

Are the conclusions drawn adequately supported by the results?

No

Are sufficient details of methods and analysis provided to allow replication by others?

Partly

Reviewer Expertise:

Injury, road traffic injuries, traumatic brain injury, health promotion, global health

I confirm that I have read this submission and believe that I have an appropriate level of expertise to confirm that it is of an acceptable scientific standard, however I have significant reservations, as outlined above.

# Response

**Published:** 2023-10-22

**Authors:** Abay Semegnew Molla

**Affiliations:** University of Gondar, Ethiopia

Thank you very much for your well informed comments.
